# Lateral hypothalamus directs stress-induced modulation of acute and psoriatic itch

**DOI:** 10.1016/j.celrep.2026.117025

**Published:** 2026-02-20

**Authors:** Jagat Narayan Prajapati, Aynal Hoque, Manojeet Pattanayak, Giriraj Sahu, Arnab Barik

**Affiliations:** 1 Centre for Neuroscience, Indian Institute of Science, Bengaluru, Karnataka 560012, India; 2 Molecular Biophysics Unit, Indian Institute of Science, Bengaluru, Karnataka 560012, India; 3 Centre for High Impact Neuroscience and Translational Applications (CHINTA), TCG CREST, Kolkata, India

## Abstract

Stress modulates itch, with acute stress suppressing and chronic stress exacerbating pruritus, yet the underlying neural mechanisms remain unclear. In this study, we investigate the role of lateral hypothalamic area (LHA) neurons in stress-induced itch modulation. Using neural-activity-dependent genetic labeling and chemogenetic tools, we selectively engaged a stress-sensitive population of LHA neurons (LHA^stress-TRAP^ neurons). Transient activation of these neurons elicited anxiety-like behavior and place aversion while suppressing both acute (chloroquine-induced) and chronic (psoriatic) itch; conversely, their inhibition potentiated itch. Notably, these neurons were not activated by acute itch but displayed activity correlated with scratching in psoriatic mice and exhibited enhanced intrinsic excitability *ex vivo*. Anterograde tracing revealed projections to key brainstem itch-modulatory regions, including the periaqueductal gray (PAG), rostral ventromedial medulla (RVM), and lateral parabrachial nucleus (LPBN). Projection-specific manipulations demonstrated that itch modulation is predominantly mediated via the PAG. Together, these findings uncover a central stress-itch circuit centered on LHA neurons and their brainstem targets.

## Graphical abstract

**Graphical abstract:**
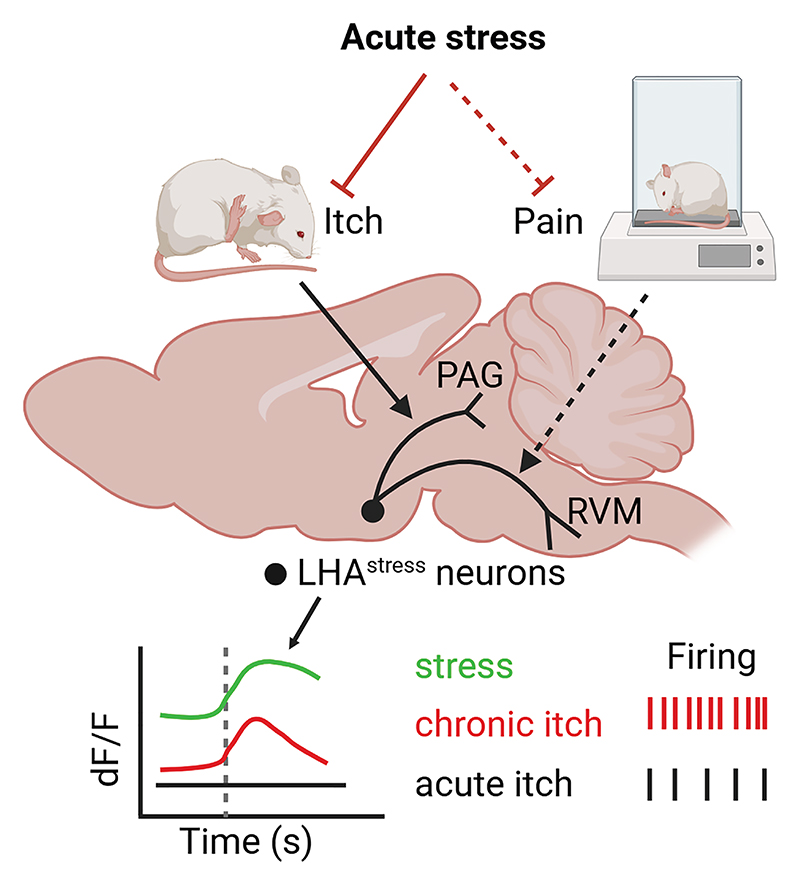

## Introduction

Itch and pain are distinct behavioral responses to noxious somatosensory stimuli: pruritic stimuli—including allergens, insect bites, and woolen textiles—elicit itch, while algesic stimuli such as noxious heat, cold, or mechanical insult evoke pain. Despite their sensory specificity, both modalities are modulated by internal brain states, including chronic stress and anxiety.^1–3^ While recent studies have elucidated key neural circuits through which stress interacts with pain perception and processing,^4–6^ the mechanisms by which stress modulates itch and how these systems may intersect remain poorly understood.

In animal models of acute stress caused by physical restraint or forced swimming, behavioral responses to algesic and pruritic chemical stimuli are suppressed.^7^ Interestingly, the extent of itch modulation was found to be proportional to the severity of stress. Similarly, acute stress attenuated mechanically (cowhage) evoked itch in human subjects.^8^ Despite these observations, the neural-circuit mechanisms underlying stress-induced modulation of itch remain unclear. Traditionally, it was thought that the central and basolateral amygdala are the primary players in stress modulation of itch.^9,10^ However, it is not known whether other brain areas play a role in the interactions between stress and itch. The lateral hypothalamic area (LHA), associated with energy homeostasis, motivated behaviors, and arousal,^11–13^ has been shown to drive stress and anxiety.^14–18^ Early lesion studies demonstrated that damage to the LHA results in diminished responsiveness to somatosensory stimuli.^19^ Recently, it was shown that excitatory LHA neurons receiving input from the anxiogenic lateral septum (LS) mediated stress-induced analgesia by suppressing pro-nociceptive neurons in the rostral ventromedial medulla (RVM).^4^ Conversely, a distinct population of inhibitory LHA neurons downstream of the LS-mediated chronic-itch-induced anxiety.^20^ The thalamic reuniens nucleus, which lies upstream of the LS-LHA circuitry, was shown to transform pruritic sensory input into anxiogenic behaviors.^20^ However, whether and how LHA neurons contribute specifically to the modulation of itch by acute stress is not known. Several brain regions that receive noxious somato-sensory input and are downstream of the LHA, including the lateral parabrachial nucleus (LPBN), periaqueductal gray (PAG), and RVM, are known to regulate pruritus and itch-related aversive behaviors. For instance, silencing LPBN neurons eliminates chloroquine-induced itch,^21–23^ while excitatory and inhibitory neurons in the ventrolateral PAG facilitate and inhibit itch,^24–26^ respectively. RVM neurons modulate pruriceptive responses through their synaptic inputs to the spinal cord.^27,28^ Thus, LHA, given its input-output connectivity and known roles in encoding aversive emotional states and the effects on determining the noxious so-matosensory thresholds, is well positioned to be a key player in the stress modulation of itch.

Here, we investigated whether neurons in the LHA mediate stress-dependent modulation of itch. To this end, we utilized the TRAP2 transgenic mouse line,^29,30^ in which tamoxifen-inducible CreERT2 is expressed under the control of the immediate-early gene *cFos* promoter, enabling activity-dependent and temporally precise genetic access to neurons via 4-hydroxytamoxifen (4-OHT) administration. By combining TRAP2 mice with locally delivered Cre-dependent adeno-associated virus (AAV) vectors, we selectively targeted the stress-activated LHA neurons (LHA^stress-TRAP^) to assess their role in chloroquine-induced acute itch and imiquimod-induced chronic psoriatic itch. Using temporally restricted chemogenetic activation and inactivation strategies, we tested whether LHA^stress-TRAP^ neurons are sufficient and necessary for itch modulation. *In vivo* calcium imaging and *ex vivo* electrophysiological recordings were employed to characterize the sensory and stress-related stimuli that recruit and alter the activity of these neurons. Additionally, anterograde and retrograde tracing approaches defined the connectivity and projection targets of LH^stress-TRAP^ neurons. Together, these circuit-level analyses demonstrate that stress-responsive LHA neurons play a key role in regulating both physiological and pathological itch.

## Results

### RS suppresses non-histaminergic acute and chronic psoriatic itch

**Figure 1:**
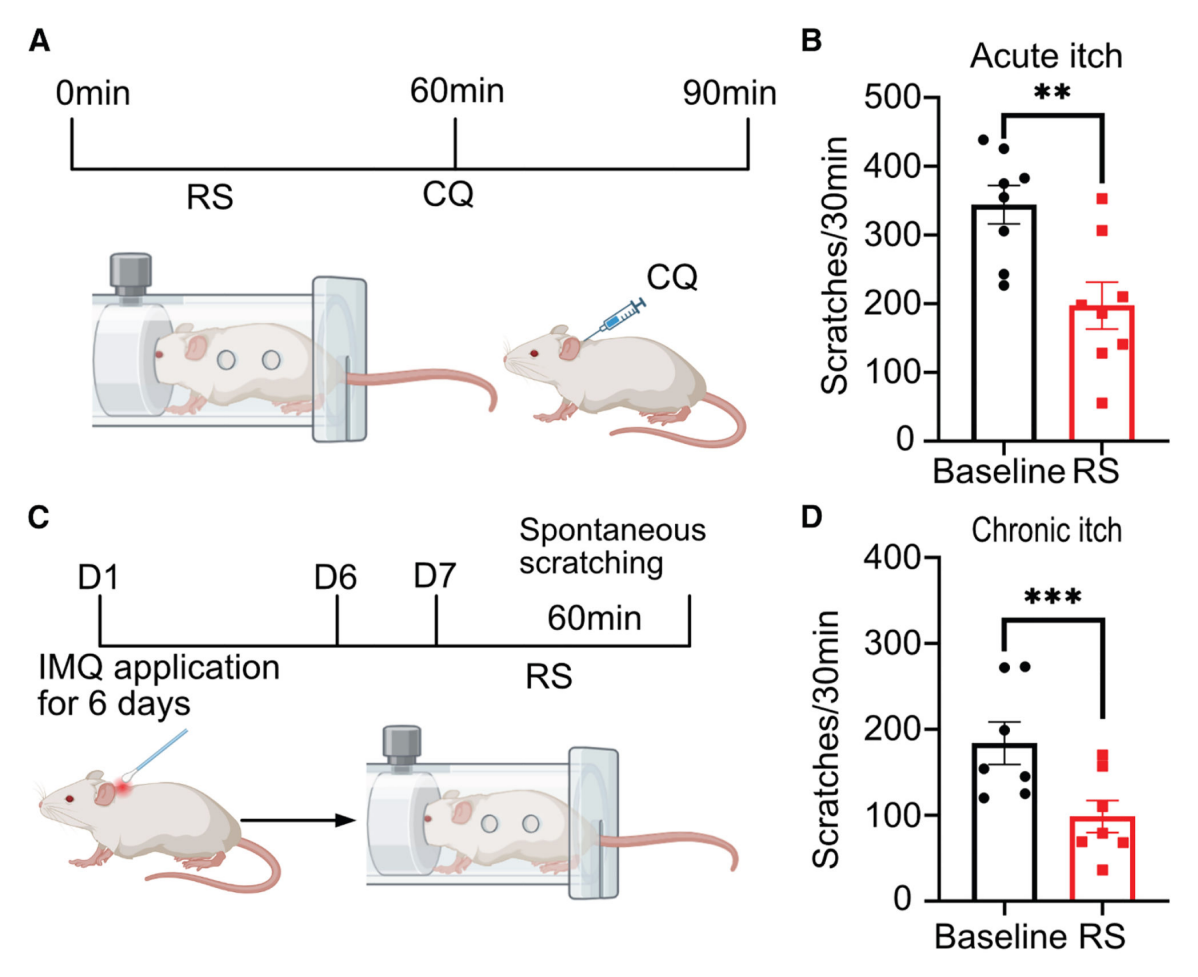
RS suppresses non-histaminergic acute and chronic psoriatic itch (A) Schematic of the experimental timeline. Mice were restrained in the restraining tube for 1 h. Intradermal chloroquine (CQ) (375 μg/75 μL) was administered into the nape of the neck, and scratching behavior was recorded for 30 min. (B) Effect of restraint stress (RS) (*t* test; 147 ± 41.31, **p = 0.0092, *n* = 8) on chloroquine-induced scratching compared with baseline scratching. (C) Schematic of the experimental timeline. Imiquimod (IMQ) was topically applied on the nape of the neck of mice to induce psoriatic itch. Mice were restrained for 1 h, and spontaneous scratching was recorded for 30 min. (D) Effect of restraint stress (RS) (*t* test; 85.57 ± 12.20, ***p = 0.0004, *n* = 7) on IMQ-induced spontaneous scratching compared with baseline spontaneous scratching.

To elucidate the neural circuits for stress modulation of itch, first, we probed whether acute restraint stress (RS) affects acute and chronic itch in mice. RS is a commonly used method to induce stress and anxiety in rodents and to study the physiological and behavioral effects of stress.^4,31–33^ We induced RS by restricting wild-type CD-1 mice in a tube for 1 h^4,31^ and injected chloroquine, a non-histaminergic pruritogen, into the nape of the neck (Figure 1A).^34^ Compared to the unrestrained control mice, RS suppressed chloroquine-induced acute itch (Figure 1B). Next, we tested whether RS suppresses the spontaneous psoriatic itch caused by repeated application of imiquimod on the nape of the neck in CD-1 mice (Figure 1C). We found that RS alleviated the pathological spontaneous itch observed in psoriatic mice compared to unrestrained animals (Figure 1D). Thus, we found that acute stress suppresses both acute and chronic itch in mice.

### LHA^stress-TRAP^ neurons are sufficient for anxiety-like behaviors in mice

**Figure 2:**
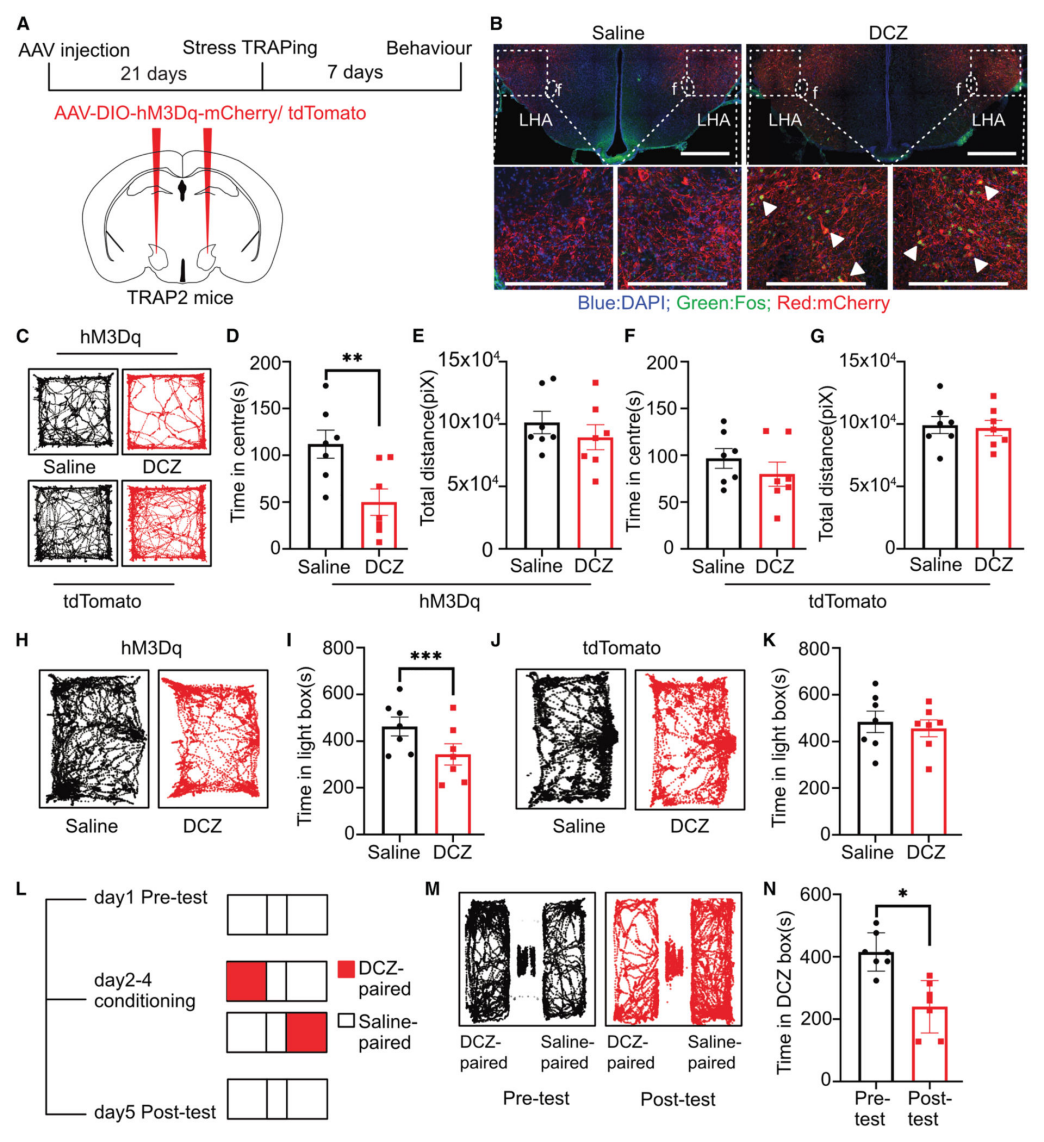
LHA^stress-TRAP^ neurons are sufficient for anxiety-like behaviors in mice (A) Schematic of the experimental timeline. AAVs encoding Cre-dependent excitatory DREADD hM3Dq or tdTomato was injected bilaterally into the LHA of TRAP2 mice. (B) Coronal-section image of LHA from the injected TRAP2 mice shows the expression of hM3Dq (red: mCherry) in LHA^stress-TRAP^ neurons. DCZ administration results in Fos (green) induction in the hM3Dq-expressing neurons. White arrowheads show yellow cells expressing both mCherry and Fos. Scale bars, 200 μm. (C) Example trajectories of mice in the open-field arena after saline/DCZ administration. (D) Effect of DCZ on time spent in the center zone (*t* test; 62.07 ± 10.52, **p = 0.0011, *n* = 7) compared with saline administration in the hM3Dq-injected mice. (E) The total distance traveled in the arena was unaffected after DCZ administration compared with saline administration in the hM3Dq-injected mice. (F) Time spent in the center zone was unaffected between DCZ- and saline-administered conditions in tdTomato-injected mice. (G) The total distance traveled in the arena was unaffected between DCZ- and saline-administered conditions in tdTomato-injected mice. (H) Example trajectory of a mouse in the light box after saline/DCZ administration in the light-dark box test. (I) Effect of DCZ on time spent in the light box (*t* test; 118.8 ± 13.49, ***p = 0.0001, *n* = 7) compared with saline administration in the hM3Dq-injected mice. (J) Example trajectory of a mouse in the light box after saline/DCZ administration in the light-dark box test in a tdTomato-injected mouse. (K) Time spent in the light box after DCZ administration was unaffected compared with saline administration in tdTomato-injected mice. (L) Experimental strategy for the conditioned place aversion (CPA) test. (M) Example trajectory of a pre- and post-conditioning mouse in the CPA apparatus. (N) Effect of DCZ on time spent in DCZ paired chamber (*t* test; 176 ± 48.05, *p = 0.0105, *n* = 7) compared with saline administration.

Next, we explored whether we could label stress-sensitive neurons in the LHA with the TRAP2 transgenic strain (Figure S1). We reasoned that the ability to carry out permanent genetic labeling of stress-sensitive neurons with Cre-recombinase in the LHA would enable transient manipulation of neural activity, anatomical mapping of pre- and postsynaptic inputs, and targeted calcium imaging. RS stress, followed by intraperitoneal (i.p.) 4-OHT administration in TRAP2 mice, stereotaxically injected with AAV-DIO-tdTomato in the LHA (Figure S1A), labeled a subpopulation of neurons with tdTomato fluorescence (Figure S1B). Reintroducing the LHA^stress-TRAP-tdTomato^ mice to RS induced cFos expression, an immediate-early gene and a proxy for neural activity in the tdTomato-expressing neurons, indicating the efficiency and the specificity in stress-TRAPping of the LHA neurons (Figure S1C). Furthermore, multiplexed *in situ* hybridizations (RNAscope) revealed that a large population of labeled neurons expressed the gene encoding the vesicular glutamate transporter, VGlut2 (*slc17a6*), suggesting that LHA^stress-TRAP^ neurons are mostly excitatory and glutamatergic (Figures S2B and S2C). Next, we tested whether the transient activation of LHA^stress-TRAP^ neurons would cause behavioral changes in mice. Specifically, we expected that stimulating LHA^stress-TRAP^ neurons would cause anxiety-like behaviors, one of the common behavioral outcomes of the RS assay. To transiently activate the LHA^stress-TRAP^ neurons, we expressed hM3Dq-mCherry, a depolarizing chemogenetic actuator, in a Cre-dependent manner (LHA^stress-TRAP-hM3Dq^) (Figure 2A). Administration of i.p. deschloroclozapine (DCZ) activated the LHA^stress-TRAP^ neurons and was reflected in the expression of cFos in the LHA^stress-TRAP-hM3Dq^ neurons expressing mCherry (fused to hM3Dq, which helps to visualize hM3Dq expression) (Figure 2B). In addition, we confirmed whether RS engages the LHA^stress-TRAP-hM3Dq^ neurons by performing RS and testing whether cFos and mCherry co-localize (Figures S3B and S3C). As expected, we found significant overlap between them. We found that DCZ in LHA^stress-TRAP-hM3Dq^ mice and not in control mice expressing tdTomato in the LHA (LHA^stress-TRAP-tdTomato^) promoted anxiety-like behaviors as assayed on the open-field test (OFT), where mice with anxiety tend to spend less time in the center zone, and on the light-dark box (LDB) test, during which anxious mice preferred to stay in the dark box. Together, our behavioral data indicate that the chemogenetic activation of the LHA^stress-TRAP^ neurons is sufficient to promote stress and anxiety (Figures 2C–2K).

Further, we determined whether transient activation of the LHA^stress-TRAP^ neurons is enough to cause learned aversion, which was tested through the conditioned place aversion (CPA) test. In the CPA test, where the conditioning stimulus is the chemogenetic activation of a neuronal population of interest, the experimental animals are paired with a chamber with i.p. DCZ and asked whether the pairing is sufficient to drive aversion. We conditioned one of the chambers of the CPA apparatus with DCZ in a cohort of LHA^stress-TRAP-hM3Dq^ mice and the other chamber with i.p. saline (Figure 2L). We found that the LHA^stress-TRAP-hM3Dq^ mice spent less time in the DCZ-paired chamber on the test day (Figures 2M and 2N). Thus, activation of LHA^stress-TRAP^ neurons is sufficient to drive CPA.

### LHA^stress-TRAP^ neurons bidirectionally control acute and chronic itch

**Figure 3:**
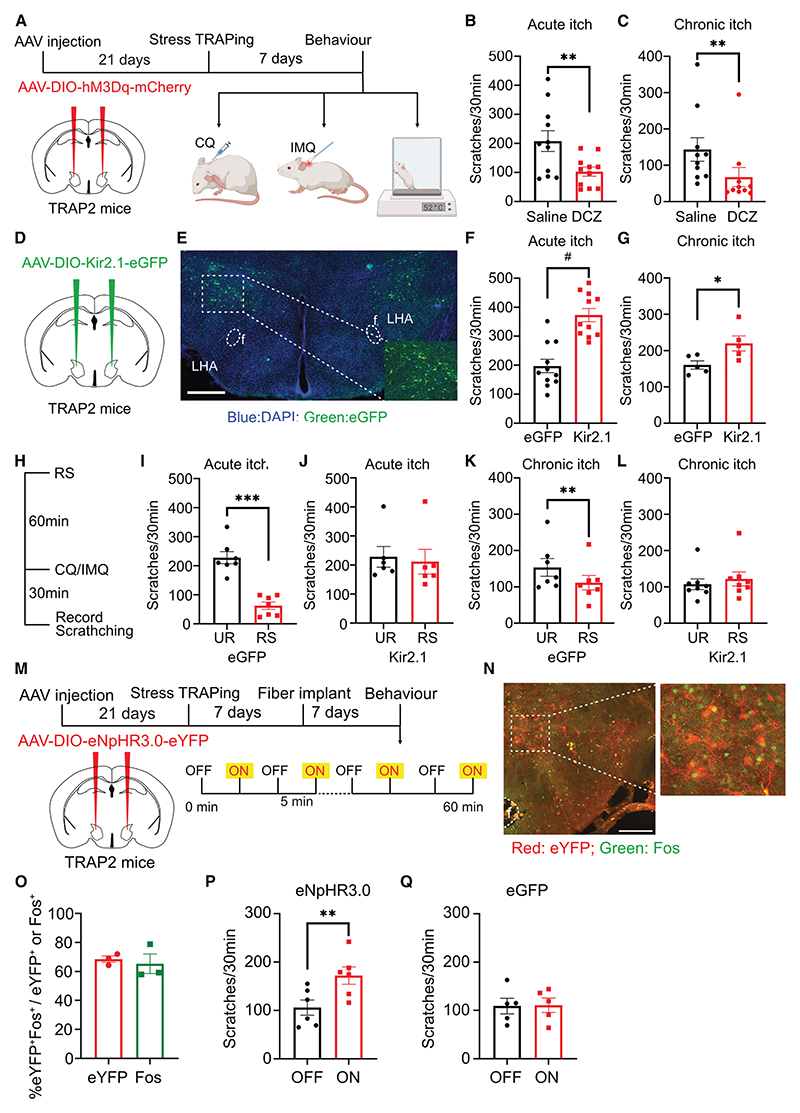
LHA^stress-TRAP^ neuron activation suppresses acute and chronic itch (A) Schematic of the experimental timeline. AAV encoding Cre-dependent excitatory DREADD, hM3Dq-mCherry, was injected bilaterally into the LHA of TRAP2 mice. (B) Effect of i.p. DCZ (*t* test; 105.2 ± 31.43, **p = 0.0074, *n* = 11) administration on chloroquine-induced scratching compared with controls injected with i.p. saline. (C) Effect of i.p. DCZ (*t* test; 76 ± 19.99, **p = 0.0042, *n* = 10) administration on psoriatic-itch-induced spontaneous scratching compared with controls. (D) AAV encoding Cre-dependent Kir2.1 was injected bilaterally into the LHA of TRAP2 mice, and the animals were stress-TRAPed after 21 days. (E) Coronal section of LHA showing the expression of Kir2.1 marked by the expression of GFP (green, DAPI-blue) in LHA^stress-TRAP^ neurons. Scale bar, 200 μm. (F) Effect of Kir2.1 silencing of LHA^stress-TRAP^ neurons (unpaired *t* test; 175.5 ± 32.49, ^#^*p* = 0.0001) on chloroquine-induced scratching compared with GFP controls. (G) Effect of Kir2.1 silencing of LHA^stress-TRAP^ neurons (unpaired *t* test; 59.20 ± 20.05, *p = 0.0184) on psoriatic-itch-induced spontaneous scratching compared with GFP controls. (H) Schematic of the experiment timeline for testing the necessity of LHA^stress-TRAP^ neurons in stress-induced suppression of itch. (I) Effect of restraint stress (RS) (*t* test; 165.4 ± 18.31, ***p = 0.0001, *n* = 7) on chloroquine-induced scratching compared with unrestrained (UR). (J) Effect of restraint stress (RS) on chloroquine-induced scratching compared with unrestrained (UR) in mice with Kir2.1-mediated silenced LHA^stress-TRAP^ neurons. (K) Effect of restraint stress (RS) (*t* test; 42 ± 9.86, **p = 0.0053, *n* = 7) on psoriatic-itch-induced spontaneous scratching compared with unrestrained (UR). (L) Effect of restraint stress (RS) on psoriatic-itch-induced spontaneous scratching compared with unrestrained (UR) in mice with Kir2.1-mediated silenced LHA^stress-TRAP^ neurons. (M) Schematic of the experiment. AAV encoding Cre-dependent eNpHR3.0 was bilaterally injected into the LHA of TRAP2 mice. One week after stress TRAPping, fiber-optic cannulas were bilaterally implanted at LHA. (N) Coronal section confocal image of the LHA^stress-TRAP^ neurons shows the overlap between eYFP-positive (red) cells and cFos-positive (green) cells. Right image shows the zoomed-in image of the marked square. Yellow cells are both positive. Scale bar, 200 μm. (O) Quantification of (N). The red bar indicates the percentage of eYFP-positive cells also expressing Fos (mean ± SD: 68.45 ± 3.19), and the green bar represents the percentage of Fos-positive cells also expressing eYFP (mean ± SD: 65.31 ± 9.56) from *n* = 3 mice (four sections per mouse). (P) Effect of light ON in LHA (*t* test; 66.33 ± 10.52, **p = 0.0015, *n* = 6) on chloroquine-induced scratching compared with light OFF in eNpHR injected mice. (Q) Light ON in LHA had no effect on chloroquine-induced scratching compared with light OFF in eGFP-injected mice.

In the next set of experiments, we tested the effect of chemogenetic activation of LHA^stress-TRAP^ neurons on acute and chronic itch (Figure 3A). As already mentioned, experimental acute itch was induced by intradermal injection of chloroquine (Figures 1A and 1B). In contrast, chronic itch was modeled by repeated application of imiquimod to the nape of the neck of mice (Figures 1C and 1D). We found that i.p. DCZ-mediated stimulation of the LHA^stress-TRAP-hM3Dq^ neurons suppressed both acute and chronic itch (Figures 3B and 3C), while the same manipulation did not affect acute and chronic itch in the tdTomato-injected control mice (Figures S4G and S4H). In addition to the chloroquine-induced itch, activation of the LHA^stress-TRAP-hM3Dq^ neurons suppressed itch induced by intradermal histamine (Figures S3D and S3E). We previously showed that the LHA neurons downstream of the LS mediate stress-induced analgesia. Hence, we wondered whether activating the LHA^stress-TRAP^ neurons enhanced spinal reflexive and supraspinal thermal pain thresholds. We used the tail-flick test to determine the spinal reflexive thermal nociceptive thresholds, and to assay the supraspinal thresholds and nocifensive behaviors we used the hot-plate assay. We found that transient activation of the LHA^stress-TRAP^ neurons increased spinal and supraspinal thermal thresholds (Figures S4A–S4E), while nociceptive thresholds remained unchanged in the control mice (Figures S4I–S4M). Thus, LHA^stress-TRAP^ neurons mediate stress-mediated suppression of pain and itch. Next, we wondered how silencing the LHA^stress-TRAP^ neurons would affect acute and chronic itch. To this end, we expressed the inwardly rectifying potassium Kir2.1 channel in the LHA^stress-TRAP^ neurons in a Cre-dependent manner (LHA^stress-TRAP-Kir2.1^) (Figures 3D and 3E). We validated neuronal silencing by Kir2.1 using calcium imaging with fiber photometry. To this end, we expressed Kir2.1-eGFP together with the genetically encoded red calcium sensor jRGECO1a in LHA^stress-TRAP^ neurons. Control mice expressed only eGFP instead of Kir2.1 (Figure S5A). We observed the co-expression of Kir2.1 and jRGE-CO1a in the LHA^stress-TRAP^ neurons (Figure S5B). As expected, Kir2.1 expression significantly reduced both baseline calcium transients (Figures S5C and S5D) and the stress-induced calcium rise during the tail-hang test compared to eGFP controls (Figures S5E–S5H). Hence, we conclude that Kir2.1 expression can effectively silence the activity of LHA^stress-TRAP^ neurons. Next, we examined the effect of silencing LHA^stress-TRAP^ neurons on itch and compared it with the eGFP controls. We injected AAV-DIO-eGFP in the LHA of stress-TRAP mice and used them as controls. Silencing the LHA^stress-TRAP^ neurons increased acute itch induced by chloroquine and imiquimod-induced psoriasis compared to control mice expressing GFP (Figures 3F and 3G). Thus, the LHA^stress-TRAP^ neurons are necessary for maintaining normal scratching frequency in response to pruritogens and chronic inflammatory itch. We then asked whether the LHA^stress-TRAP^ neurons are required for stress-induced suppression of acute and chronic itch. We found that Kir2.1-mediated silencing of the LHA^stress-TRAP^ neurons reduced the suppressive effects of RS on acute and psoriatic itch (Figures 3J and 3L). At the same time, in GFP-expressing control mice, RS inhibited itch, as seen previously (Figures 3I and 3K). In conclusion, the LHA^stress-TRAP^ neurons are required for acute-stress-mediated itch suppression.

Since Kir2.1 causes permanent neuronal silencing, it cannot be used to study the effect of transient and reversible silencing of LHA^stress-TRAP^ neurons on itch. Transient silencing may reveal the acute necessity of LHA^stress-TRAP^ neurons in itch. We therefore used halorhodopsin (eNpHR3.0), an inhibitory optogenetic actuator, by expressing it in LHA^stress-TRAP^ neurons to silence the neurons transiently with a yellow laser (595 nm) (Figures 3M–3O). We confirmed the eNpHR3.0-YFP-expressing LHA^stress-TRAP^ neurons to be sensitive to stress by performing RS and probing for cFos expression (Figures 3N and 3O). As observed with Kir2.1-mediated irreversible silencing of the LHA^stress-TRAP^ neurons, transient inhibition increased scratching in response to chloroquine (Figure 3P). This effect was specific, as the yellow laser stimulation did not alter scratching in control mice expressing eGFP (Figure 3Q). Altogether, we found that the LHA^stress-TRAP^ neurons bidirectionally control acute and chronic itch.

### Input-output mapping of LHA^stress-TRAP^ neurons

The advent of virally mediated anatomic tracing techniques has revolutionized the mapping of inputs and outputs of select neurons in the central nervous system. Hence, the monosynaptic rabies tracing technique was used here to delineate the presynaptic inputs in the brain. For the monosynaptic rabies tracing, we stereotaxically delivered Cre-dependent AAVs carrying the genes for the TVA-GFP and RVΔG in the LHA of TRAP2 mice. After 3 weeks, we trapped the stress-sensitive neurons in the LHA with 4-OHT to enable TVA-GFP and RVdelG expression in LHA^stress-TRAP^ neurons. One week later, we injected RV-N2C-delG-nlstdTomato into the LHA (Figure S6A). We found that within a week, LHA^stress-TRAP^ neurons express the TVA-tagged GFP (green) and tdTomato (red) from the modified rabies virus, indicating that the double-fluorescently labeled yellow cells are the starter cells (Figure S6C). We observed neurons with red fluo-rescence in the pruriceptive brain areas such as the LPBN, PAG, and RVM; somatosensory information processing areas such as the deeper layers of the primary somatosensory cortex (S1); and affective-motivational brain nuclei such as the locus coeruleus, basolateral amygdala, bed nucleus of stria terminalis, LS, and medial septum (Figures S6D and S6E). Previously, we found that the inhibitory neurons in the LS project to the LHA to drive RS-induced analgesia.^4^ Since we found that the LS and the LHA^stress-TRAP^ neurons are monosynaptically connected, we tested whether the LHA^stress-TRAP^ neurons are the same as the LHA_post-LS_ cells. To this end, we injected the anterograde trans-synaptic AAVTranssyn-FlpO in the LS of TRAP2 mice, facilitating the availability of FlpO recombinase specifically in the LHA_post-LS_ neurons. Simultaneously, we delivered DIO-GFP and fDIO-tdTomato to the LHA (Figure S8A). After stress trapping, the LHA_post-LS_ and the LHA^stress-TRAP^ neurons expressed tdTomato and GFP, respectively. We found that a few of the GFP- and tdTomato-expressing neurons overlap (Figures S8B and S8C). This implies that the majority of the LHA^stress-TRAP^ neurons do not receive input from the LS. This is in line with our previous findings, where the inhibitory neurons were engaged by RS and suppressed LHA activity.^4^ Together, unbiased retrograde mapping of monosynaptic inputs implies that the negative-affective signals due to RS might be routed through the forebrain and midbrain limbic areas or brainstem nuclei encoding aversive sensory stimuli.

Next, we sought to map the brain-wide axonal projections of LHA^stress-TRAP^ neurons. To this end, we injected cell-filling AAV-DIO-tdTomato into the LHA of TRAP2 mice and stress-TRAPed after 3 weeks (Figure S7A). We observed robust expression of tdTomato in the LHA^stress-TRAP^ neurons (Figure S7B). Whole-brain sectioning and confocal imaging revealed LHA^stress-TRAP^ projections in diverse brain areas, including the infralimbic cortex, various subnuclei of the septum, bed nucleus of stria terminalis, central amygdala, paraventricular thalamus, and lateral habenula. In the brainstem, it projects to the PAG, ventral tegmental area (VTA), parabrachial nucleus (PBN), RVM, and reticular formation (Figures S7C and S7D). Intriguingly, through anterograde and retrograde tracing experiments, we realized that the LHA^stress-TRAP^ neurons have reciprocal connections with brain nuclei such as the central amygdala, LPBN, and RVM.

### The activity of LHA^stress-TRAP^ neurons coincides with spontaneous scratching in psoriatic conditions

**Figure 4:**
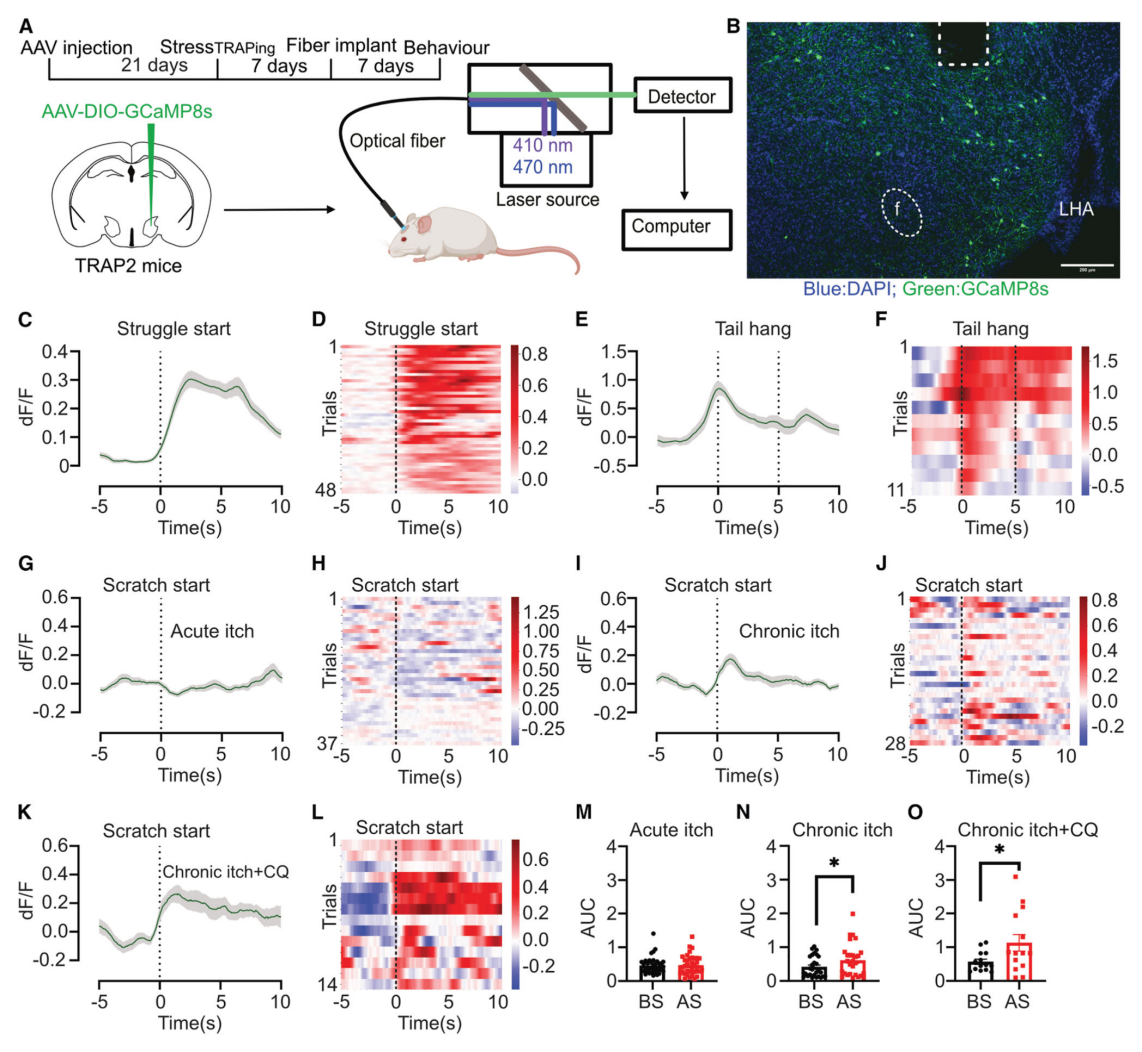
LHA^stress-TRAP^ neurons respond to chronic-itch-induced scratching (A) Schematic of the experiment. AAV encoding Cre-dependent GCaMP8s was injected into the LHA of TRAP2 mice. The right panel shows the schematics of dual-color photometry. (B) Coronal section image of LHA from the injected TRAP2 mice shows the expression of GCaMP8s (green; DAPI: blue) in LHA^stress-TRAP^ neurons. The white dotted line shows the fiber track. Scale bar, 200 μm. (C) The average fluorescent signal at the start of the struggle in the restraint stress. (D) Heatmap showing the response to struggle in the restraint stress test. (E) The average fluorescence signal during the tail-hang test. (F) Heatmap showing the response during the tail-hang test. (G) The average fluorescence signal at the start of chloroquine-induced scratching. (H) Heatmap showing the response at the start of chloroquine-induced scratching. (I) The average fluorescence signal at the start of spontaneous scratching in psoriatic chronic itch. (J) Heatmap showing the response of spontaneous scratching in psoriatic chronic itch. (K) The average fluorescence signal at the start of scratching induced by chloroquine in psoriatic chronic itch. (L) Heatmap showing the response of scratching induced by chloroquine in psoriatic chronic itch. (M) Area under the curve (AUC) 5 s before and after the start of chloroquine-induced scratching. (N) AUC 5 s before and after the start of spontaneous scratching in chronic itch (*t* test; 0.1993 ± 0.08799, *p = 0.0318, *n* = 28). (O) AUC 5 s before and after the start of chloroquine-induced scratching in chronic itch (*t* test; 0.5633 ± 0.2047, *p = 0.0165, *n* = 14).

Genetically encoded fluorescent calcium sensors, such as GCaMP6s, and *in vivo* calcium imaging techniques, such as fiber photometry, have revolutionized *in vivo* monitoring of neural activity in behaving mice. Here, we tested the activity of LHA^stress-TRAP^ neurons when the mice were under RS. We injected the AAV9-DIO-GCaMP8s in the LHA of TRAP2 mice and expressed GCaMP8s in the acute-stress-sensitive neurons by i.p. administration of 4-OHT before exposing the mice to the RS assay (Figure 4A). Next, we implanted a fiber-optic cannula (200 μm inner diameter) in the LHA so that the fluorescent dynamics of the GCaMP8s sensor expressed in the LHA^stress-TRAP^ neurons could be recorded with a fiber photometry setup (Figure 4A, right panel). Predictably, we found that the LHA^stress-TRAP^ neurons were engaged when the mice were restrained, and specifically, the rise in GCaMP8s fluorescence levels coincided with the bouts of struggle (Figures 4C and 4D). Similarly, when mice were hung by their tails, a commonly used mild stress stimulus, the LHA^stress-TRAP^ neurons were active (Figures 4E and 4F). However, despite the inhibitory effects of the LHA^stress-TRAP^ neurons on acute and chronic itch (Figure 3), the activity of these neurons did not coincide with scratching bouts induced by chloroquine (Figures 4G, 4H, and 4M). Similarly, the activity of the LHA^stress-TRAP^ neurons did not coincide with nocifensive behaviors, such as licks and shakes, on the 52°C thermal-plate test (Figures S9A–S9F). The thermal-plate test enables testing of rodent behavior exposed to a range of surface temperatures, hot and cold, at both innocuous and noxious ranges. Exposed to unbearable, noxious heat above 44°C, mice and rats respond with reflexive shaking and coping licking responses. Thus, the LHA^stress-TRAP^ neurons are tuned to the stimuli with the potential to cause stress and anxiety; however, they are not activated while mice experience and react to aversive and noxious somatosensory stimuli causing itch and pain. Next, we tested whether the activity of LHA^stress-TRAP^ neurons is altered in psoriatic conditions and whether the pathological spontaneous scratching correlates with the neural activity (Figures 4I, 4J, and 4N). To our surprise, we found that neural activity in the LHA^stress-TRAP^ cells correlated with spontaneous (alloknesis) and chloroquine-evoked (hyperkinesis)^35^ scratching in mice with imiquimod-induced psoriasis (Figures 4K, 4L, and 4O). This finding is in contrast to our observation that the activity of LHA^stress-TRAP^ neurons does not coincide with chloroquine-induced acute scratching (Figures 4G and 4H). Thus, through fiber-photometry recordings, we found that the LHA^stress-TRAP^ neurons are activated by stressors and engaged by itch under chronic psoriatic conditions.

### LHA^stress-TRAP^ neurons display potentiated excitability in psoriatic mice

**Figure 5:**
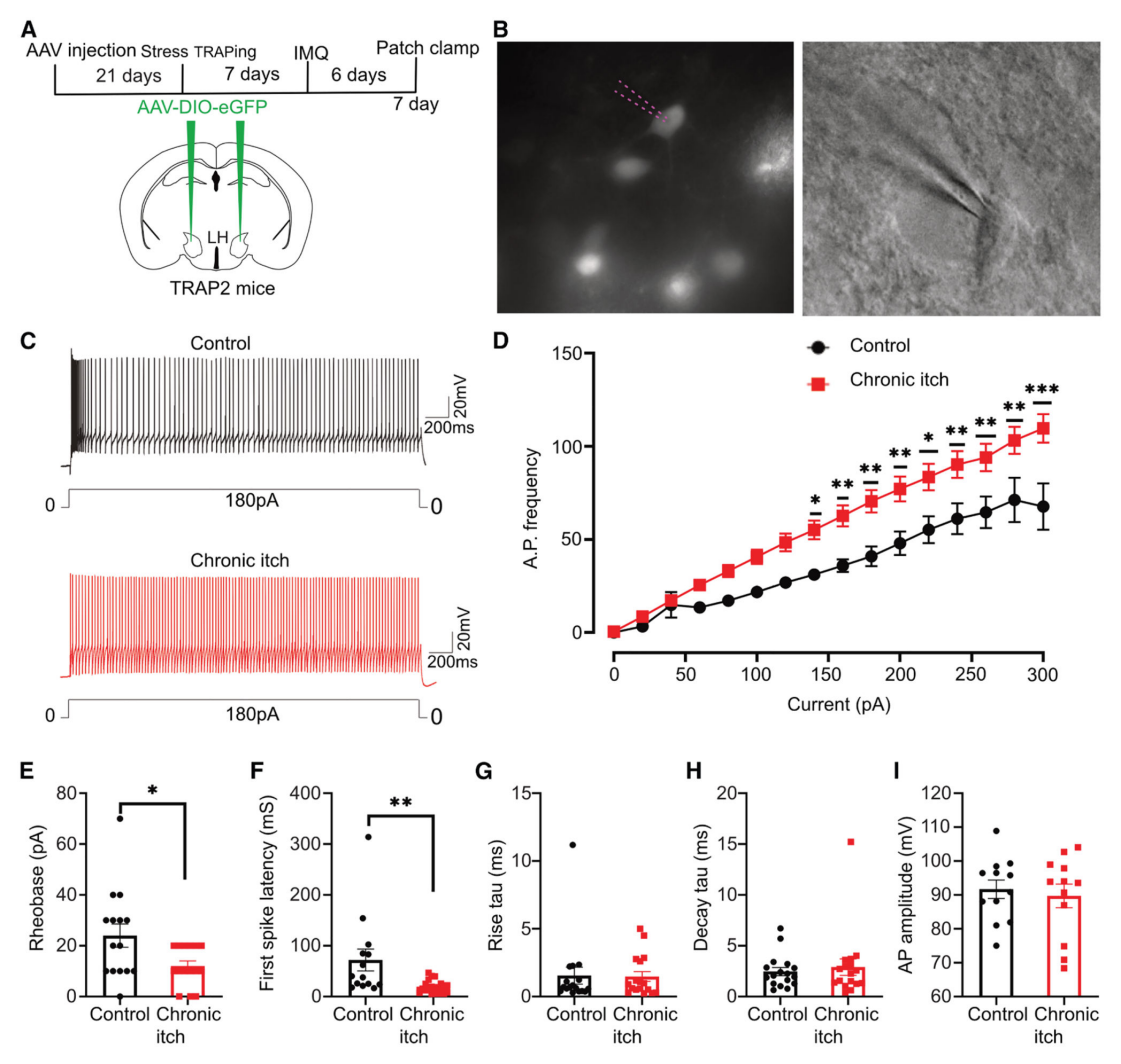
LHA^stress-TRAP^ neurons are rendered hyperexcitable by psoriatic itch (A) Schematic of the experimental timeline. AAV encoding Cre-dependent GFP was injected bilaterally into the LHA of TRAP2 mice. (B) Image of a representative patched cell. Dotted lines show the pipette tip. (C) Representative trace of AP firing in control and chronic itch mice from the LHA^stress-TRAP^ neurons. (D) Effect of chronic itch on the frequency of AP compared with control AP mice. Two-way ANOVA (*n* = 17 cells for chronic itch from six mice and *n* = 12 cells from five control mice, *p* values from 140-to 300-pA currents are 0.0274, 0.008, 0.0071, 0.0053, 0.0122, 0.0086, 0.0081, 0.0054, and 0.0001). (E) Effect of chronic itch on rheobase (unpaired *t* test; 12 ± 4.976, *p = 0.0227) of the LHA^stress-TRAP^ neurons compared with controls. (F) Effect of chronic itch on first spike latency of the LHA^stress-TRAP^ neurons (unpaired *t* test; 52.84 ± 18.66, **p = 0.0081). (G) Chronic itch had no effect on the rise in tau of the LHA^stress-TRAP^ neurons compared with controls. (H) Chronic itch had no effect on the decay tau of the LHA^stress-TRAP^ neurons compared with controls. (I) Chronic itch had no effect on the action potential amplitude of the LHA^stress-TRAP^ neurons compared with controls.

*In vivo* recordings revealed that LHA^stress-TRAP^ neurons become responsive to pruritic stimuli in mice that develop experimental psoriasis (Figures 4K, 4L, and 4O). Thus, we hypothesized that imiquimod may sensitize LHA^stress-TRAP^ neurons, which should be reflected in increased firing rates in *ex vivo* preparations with postsynaptic current injections. To test this, we performed whole-cell patch-clamp electrophysiological recordings in fluo-rescently labeled (eGFP) LHA^stress-TRAP^ neurons from mice with and without psoriasis (controls) (Figures 5A and 5B). We expressed eGFP in LHA^stress-TRAP^ neurons using the method described in previous sections (Figure S1). Indeed, we observed a gain in firing in LHA neurons of mice with imiquimod-induced psoriasis as compared to neurons in control mice, reflected in the *F*/*I* (frequency/current) plot by an increase in the frequency of action potentials upon a series of injections (Figure 5D) with postsynaptic depolarizing current ranging from 0 to 300 pA in 20-pA increments. Similarly, we observed a significant hyperpo-larizing shift in the rheobase membrane voltage, implying higher excitability propensity of LHA^stress-TRAP^ neurons of psoriasis mice compared to the controls (Figure 5E). Furthermore, the latency to first spike firing had decreased in the psoriasis mice compared to the controls (Figure 5F). Meanwhile, the action potential amplitude, rise, and decay time constants remained unaffected between psoriasis and control neurons (Figures 5G–5I). Notably, the passive membrane properties, such as input resistance and membrane voltage, did not change between the psoriasis and control mice (Figures S10A and S10B), indicating an intrinsic change in membrane excitability contributing to the higher excitability of LHA^stress-TRAP^ neurons in psoriasis mice.

### LHA^stress-TRAP^ neurons suppress acute and chronic itch through axonal projections in the PAG and RVM

**Figure 6:**
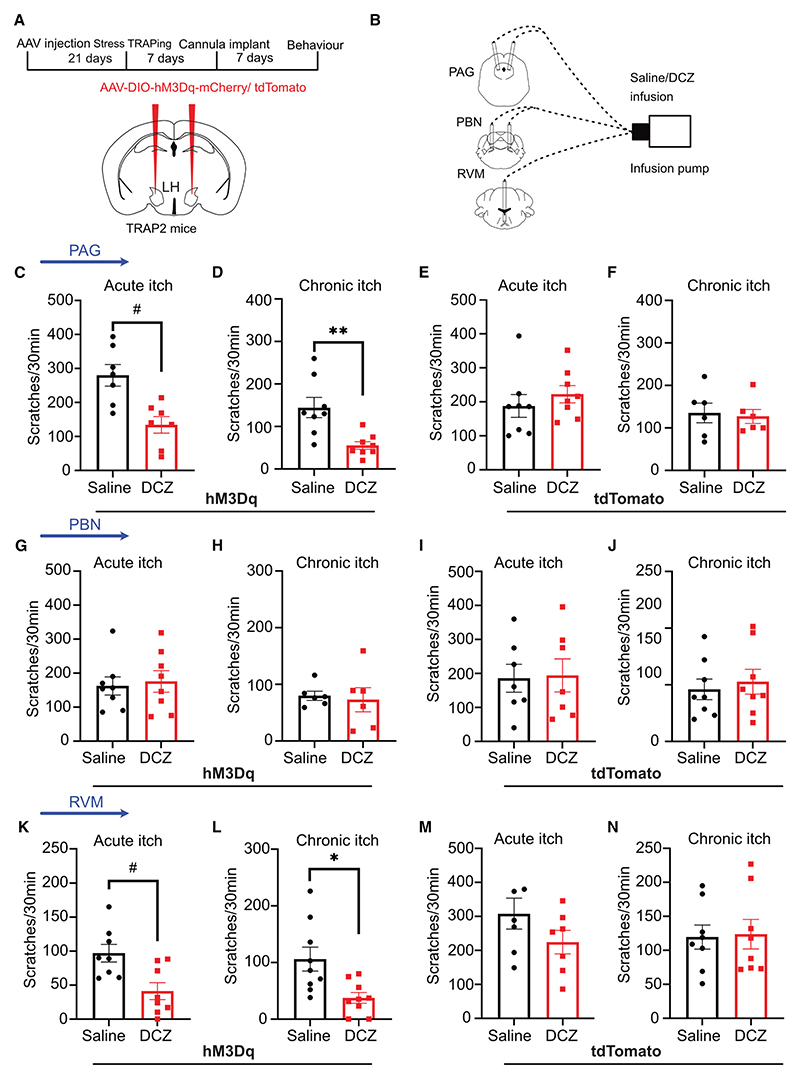
LHA^stress-TRAP^ neurons suppress acute and chronic itch through axonal projections in the PAG and RVM (A) Schematic of the experimental timeline. AAV encoding Cre-dependent excitatory DREADD hM3Dq was injected bilaterally into the LHA of TRAP2 mice. One week after stress-TRAPping, bilateral cannulas were implanted at the ventrolateral PAG, PBN, and RVM. (B) Schematics of DCZ/saline infusion at PAG, PBN, and RVM. (C) Effect of DCZ infusion at PAG (*t* test; 145.9 ± 13.97, ^#^*p* < 0.0001, *n* = 7) on chloroquine-induced scratching compared with saline infusion. (D) Effect of DCZ infusion at PAG (*t* test; 89.50 ± 17.02, **p = 0.0012, *n* = 8) on psoriatic-itch-induced spontaneous scratching compared with saline infusion. (E) DCZ infusion at PAG has no effect on chloroquine-induced scratching compared with saline infusion in tdTomato-injected control mice. (F) DCZ infusion at PAG on psoriatic itch induced spontaneous scratching compared with saline infusion in tdTomato-injected control mice. (G) DCZ infusion at PBN has no effect on chloroquine-induced scratching compared with saline infusion. (H) DCZ infusion at PBN has no effect on psoriatic-itch-induced spontaneous scratching compared with saline infusion. (I) DCZ infusion at PBN has no effect on chloroquine-induced scratching compared with saline infusion in tdTomato-injected control mice. (J) DCZ infusion at PBN has no effect on psoriatic-itch-induced spontaneous scratching compared with saline infusion in tdTomato-injected control mice. (K) Effect of DCZ infusion at RVM (*t* test; 55.75 ± 6.761, ^#^*p* < 0.0001, *n* = 8) on chloroquine-induced scratching compared with saline infusion. (L) Effect of DCZ infusion at RVM (*t* test; 68.56 ± 21.86, *p = 0.0139, *n* = 9) on psoriatic-itch-induced spontaneous scratching compared with saline infusion. (M) DCZ infusion at RVM has no effect on chloroquine-induced scratching compared with saline infusion in tdTomato-injected control mice. (N) DCZ infusion at RVM has no effect on psoriatic-itch-induced spontaneous scratching compared with saline infusion in tdTomato-injected control mice.

LHA^stress-TRAP^ neurons were sufficient to suppress acute and chronic psoriatic itch (Figure 3). Hence, we sought to understand the downstream target brain nuclei through which LHA^stress-TRAP^ neurons mediate the expression of nocifensive scratching responses to pruritic stimuli. Anterograde tracing revealed that LHA^stress-TRAP^ neurons project to regions such as the PAG, PBN, and RVM, which are known to modulate itch-induced scratching through direct or indirect projections to the spinal cord (Figure S7). Notably, these brainstem nuclei are interconnected, receive hypothalamic inputs, and are known to determine modulation of nociceptive and itch thresholds by internal brain states such as stress and hunger.^4,36–38^ We expressed hM3Dq-mCherry in LHA^stress-TRAP^ neurons (LHA^stress-TRAP-hM3Dq^) using the genetic strategy described in Figure 2 and implanted cannulas in the axonal target regions, such as the PAG, LPBN, and RVM, to enable localized DCZ infusion and specific excitation of the downstream neurons. We implanted bilateral cannulas over the PAG and LPBN and single cannulas over the RVM due to the central location of the nuclei. Successful expression of hM3Dq-mCherry in the LHA^stress-TRAP^ neurons and labeling of axon terminals in the target regions was evidenced by the presence of mCherry (red) terminals. Meanwhile, chemogenetic activation of PAG, LPBN, and RVM target neurons by DCZ infusion was confirmed by cFos expression (Figure S11). As controls for the targeted chemogenetic activation of LHA^stress-TRAP^ downstream target neurons, we expressed tdTomato in LHA^stress-TRAP^ (LHA^stress-TRAP-tdTomato^) neurons. Thus, in the control mice, DCZ infusion in the LHA^stress-TRAP-tdTomato^ mice would not activate neurons in the LHA, PAG, LPBN, or RVM (Figure S12). We found that DCZ infusion through cannulas in the PAG and RVM of LHA^stress-TRAP-hM3Dq^ mice, but not in the LPBN, suppressed intradermal chloroquine-induced itch in the nape of the neck (Figures 6C–6G and 6K). Since chemogenetic activation of the LHA^stress-TRAP^ neurons resulted in analgesia on the hot-plate and tail-flick tests, and LHA-RVM circuitry is known to modulate nociceptive thresholds, we tested whether DCZ infusion in the RVM of the LHA^stress-TRAP-hM3Dq^ mice would affect nocifensive behaviors to thermal stimuli. We found that transient activation of LHA^stress-TRAP^ terminals in the RVM was sufficient to increase the latency to lick and shake on the hot-plate test, increasing the frequency of occurrences of the nocifensive behaviors and resulting in elevated thresholds on the tail-flick test (Figures S13C–S13G), while the same manipulation did not affect the pain thresholds in the control LHA^stress-TRAP-tdTomato^ mice (Figures S13H–S13L). Similarly, In LHA^stress-TRAP-tdTomato^ mice, DCZ at the PAG, LPBN, or RVM did not alter scratching by chloroquine (Figures 6E–6I and 6M). At the same time, spontaneous scratching caused by repeated application of imiquimod was suppressed by chemogenetic activation of axon terminals of the LHA^stress-TRAP-hM3Dq^ neurons in the PAG and RVM but not the LPBN (Figures 6D–6H and 6L). DCZ infusion in either of the downstream targets of the LHA^stress-TRAP^ neurons in tdTomato-expressing mice did not alter psoriasis-induced spontaneous scratching (Figures 6F–6J and 6N). Thus, LHA^stress-TRAP^ neurons, through their downstream targets in the PAG and RVM, mediate the effects of stress on acute and chronic itch.

**Figure 7:**
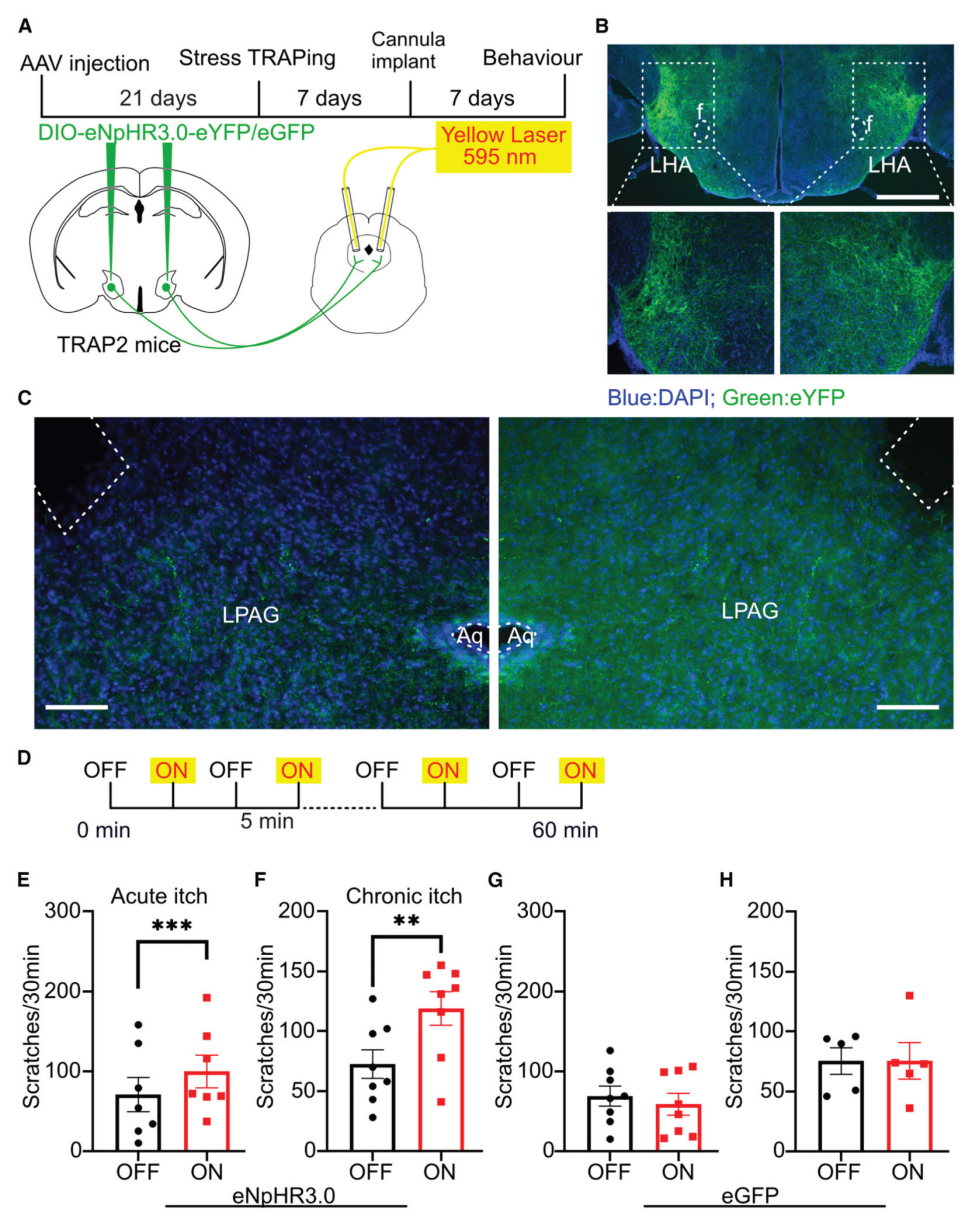
Inhibition of LHA^stress-TRAP^ neurons’ PAG terminals exacerbate acute and chronic itch (A) Schematic of the viral injection of enhanced *Natronomonas pharaonis* halorhodopsin (eNpHR) to inhibit the PAG terminals of LHA^stress-TRAP^ neurons. (B) Coronal-section image of LHA showing the expression of eNpHR in the LHA^stress-TRAP^ neuron, marked by the expression of eGFP (green; DAPI, blue). A higher-resolution image of the inset squares is shown at the bottom. Scale bar 200 μm. (C) Coronal-section image of PAG showing the axon terminals of the LHA^stress-TRAP^ neurons. White dashed lines show fiber tracks. Scale bars, 200 μm. (D) Schematic of optogenetic stimulation. (E) Effect of light ON in PAG (*t* test; 29 ± 4.796, ***p = 0.0009, *n* = 7) on chloroquine-induced scratching compared with light OFF in eNpHR-injected mice. (F) Effect of light ON in PAG (*t* test; 46.50 ± 10.01, **p = 0.0024, *n* = 8) on psoriatic-itch-induced spontaneous scratching compared with light OFF in eNpHR-injected mice. (G) Light ON in PAG had no effect on chloroquine-induced scratching compared with light OFF in eGFP-injected mice. (H) Light ON in PAG had no effect on psoriatic-itch-induced spontaneous scratching compared with light OFF in eGFP-injected mice.

Further, we tested whether optogenetic inhibition of the axon terminals of LHA^stress-TRAP^ neurons in the PAG altered acute and chronic itch. To this end, we expressed halorhodopsin fused with YFP (eNpHR3.0-YFP)^39^ in a Cre-dependent manner in LHA^stress-TRAP-eNpHR3.0^ neurons with AAV vectors delivered stereotaxically following the strategy described in Figure 7A. We expressed YFP in the LHA^stress-TRAP^ neurons and used them as controls. The expression of eNpHR in the LHA^stress-TRAP^ neurons was confirmed by the expression of YFP, which was fused to eNpHR (Figure 7B). To optogenetically inhibit LHA^stress-TRAP-eNpHR3.0^, we implanted bilateral optic fiber cannula in the PAG of mice (Figure 7C). Yellow light (595 nm) shining through the cannulas in PAG exacerbated chloroquine-induced acute and imiquimod-induced chronic itch (Figures 7E and 7F). Predictably, in control experiments, yellow light shining at the YFP-expressing terminals of LHA^stress-TRAP^ neurons in the PAG did not alter acute and chronic itch (Figures 7G and 7H). In sum, the suppression of LHA^stress-TRAP^ inputs to the PAG enhances the urge to scratch the site of itch under acute and chronic pathological conditions.

## Discussion

The relationship between stress and itch is complex. Here, we shed light on the neural circuitry between the LHA and its downstream neurons in the stress modulation of acute and pathological itch. Immediate-early gene promoter-mediated genetrapping strategies allowed us to genetically label restraint stress-sensitive neurons in the LHA. These LHA^stress-TRAP^ neurons were sufficient to suppress acute and chronic itch (Figure 3). Further, these neurons were necessary for acute-stress-mediated itch suppression (Figure 3). We mapped the inputs and outputs to find that the LHA^stress-TRAP^ neurons receive somatosensory and affective-motivational inputs from various cortical and subcortical structures and send outputs to them (Figures S6 and S7). Next, the activity of LHA^stress-TRAP^ neurons corresponded to bouts of spontaneous scratching after mice had developed psoriasis and can be explained by altered cellular electrophysiological properties (Figures 4 and 5). We tested through which downstream target LHA^stress-TRAP^ neurons impart their effect on acute and chronic itch. We focused on the brain-stem structures PAG, LPBN, and RVM and found that the LHA-PAG circuitry is sufficient and necessary for the effects of acute RS on itch (Figures 6 and 7).

Chronic stress is known to exacerbate both acute and chronic itch, whereas the effects of acute stress on itch remain inconsistent across studies.^40,41^ Importantly, whether neurons in the LHA contribute to these effects has not been addressed. We found that LHA^stress-TRAP^ neurons are mostly glutamatergic (Figure S2). These glutamatergic LHA neurons were activated by stress and shown to be involved in stress modulation of feeding behaviors.^42–44^ Our data show that chemogenetic activation of LHA^stress-TRAP^ neurons induces anxiety and learned aversion (Figure 2), in agreement with previous findings.^45–48^ Acute activation of LHA glutamatergic neurons is aversive in nature and causes termination of feeding. LHA^stress-TRAP^ neurons mediated anxiety and aversion, likely through their projections to VTA or lateral habenula (Figure S7).^49,50^ Next, we showed that acute stress or activation of LHA neurons sensitive to acute stress is sufficient for suppressing both physiological and pathological itch (Figure 3). It remains to be tested whether repeated activation of the LHA^stress-TRAP^ neurons is sufficient to cause chronic stress and anxiety, as well as the resultant exacerbation of itch. Interestingly, we find that the LHA^stress-TRAP^ neurons are potentiated by psoriasis (Figure 5), and the activity of LHA^stress-TRAP^ neurons corresponds to scratching bouts in chronic itch but not in acute itch.

The contrast between how the stress-sensitive LHA neurons are engaged by acute and chronic itch may arise from two possibilities: (1) chronic psoriatic itch causes persistent stress and, hence, the LHA stress-sensitive neurons are activated; and (2) peripheral inflammation increases stress and recruits the LHA stress-sensitive neurons. By contrast, acute non-histaminergic itch induced by peripheral application of chloroquine likely failed to activate the LHA stress-sensitive neurons, as this may not be sufficient to cause inflammation or stress.

Inflammatory chronic itch conditions, such as psoriasis, can engage brain areas known to respond to elevated cytokines.^51–54^ In psoriatic mice, where the LHA^stress-TRAP^ neurons start responding to scratching, the information regarding the inflammation leading to psoriasis can likely be transmitted to the LHA through the paraventricular nucleus (PVN)^55^ (Figure S6), thus increasing the excitability of the LHA^stress-TRAP^ neurons. Alternatively, the sustained activity in the brain nuclei presynaptic to the LHA^stress-TRAP^ neurons that receive somatosensory inputs such as S1, LPBN, and anterior insular cortex under psoriatic conditions (Figure S6) can potentiate LHA^stress-TRAP^ neurons. In sum, LHA^stress-TRAP^ neurons can become sensitive to pruritic stimuli in mice with chronic itch due to increased sustained spontaneous activity in the somatosensory circuits carrying pruritic information or brain circuits engaged by a heightened immune system.

The lateral hypothalamus projects to a wide array of brain regions across the rostrocaudal axis (Figure S7).^12^ Moreover, our data suggest that stress-sensitive LHA neurons modulate itch via their synaptic connections with the PAG and RVM (Figures 6 and 7). Both of these target nuclei have been shown to modulate itch.^24–28,56,57^ Activation of the inhibitory PAG neural population has been shown to suppress itch, while inhibition enhances itch.^24,25^ However, it was not known whether PAG is involved in stress-induced modulation of itch; hence, we focused on the PAG. Since LHA^stress-TRAP^ neurons are glutamatergic in nature, it is likely that these neurons are synapsing on the PAG GABAergic neurons to bidirectionally modulate physiological and pathological itch. PAG inhibitory neurons might suppress itch through projections to the RVM. In that case, the RVM will be the common substrate through which stress-relevant top-down modulation of pain and itch occurs. Further, given the fact that both PAG and RVM terminal activation of LHA^stress-TRAP^ neurons is able to suppress itch, it will be interesting to test whether the same axon collateral projects to these nuclei to suppress itch or whether two distinct populations of glutamatergic LHA^stress-TRAP^ neurons are involved in itch modulation. Viral-mediated intersectional genetic tools can facilitate this endeavor.

At first glance, it may appear that the LHA^stress-TRAP^ neurons are independently involved in stress and itch modulation (Figures 2, 3, and 4). However, our findings suggest otherwise. Specifically, (1) acute itch does not activate LHA^stress-TRAP^ neurons (Figure 4G), (2) LHA^stress-TRAP^ neurons are engaged during exposure to anxiogenic or mildly stressful stimuli (Figures 4C–4F), and (3) activating these neurons increases anxiety while suppressing itch (Figures 2C–2K). Together, these observations indicate that LHA^stress-TRAP^ neurons are more likely involved in the stress-dependent modulation of itch rather than directly modulating itch itself. Furthermore, inhibition of LHA^stress-TRAP^ neurons abolishes stress-induced suppression of itch and increases scratching (Figures 3H–3L), which may reflect their normal role in regulating pruritogen-induced scratching through downstream periaqueductal targets. Under basal conditions, LHA^stress-TRAP^ neurons may suppress or inhibit scratching; thus, when the activity of the neurons is suppressed, the inhibition is withdrawn, and chloroquine-induced scratching is exacerbated. Thus, LHA^stress-TRAP^ neurons may simultaneously contribute to both stress modulation of itch and direct itch regulation. In that case, the two functions will be interdependent and cannot be viewed in isolation.

Dysregulation of the hypothalamic-pituitary-adrenal axis is a hallmark of psoriasis.^58,59^ The affected individuals have difficulties in coping with stress and thus are more prone to anxiety and panic attacks.^1^ A subpopulation of neurons in the LHA expressing orexin has been implicated in regulating corticosterone release through inputs to the PVN.^14^ Given the anatomical location of the LHA^stress-TRAP^ neurons and the fact that these neurons are glutamatergic and project to the PAG (Figure S7), they likely co-express orexin.^60^ Thus, LHA^stress-TRAP^ neurons and downstream circuitry can be potentially involved in the stress-coping pathophysiology observed in psoriatic patients.^61^

The caveat of leveraging the Fos-TRAP strategy to study functionally relevant neural populations is that it does not take into account the progress made in dissecting the LHA population according to assigned classes defined by their molecular markers. Glutamatergic Esr1-expressing LHA neurons projecting to the lateral habenula mediate the development of a sex-specific stress state,^62^ while VTA-projecting glutamatergic LHA neurons are potentiated by stress, which regulates dopamine release in the prefrontal cortex, thereby promoting stress eating.^43^ The midbrain dopaminergic system is known to mediate key aspects of scratch initiation and termination in acute itch^63–66^; thus, LHA-VTA connections can mediate stress-itch interactions. Parvalbumin^+^ glutamatergic projection neurons with synaptic connections with PAG in the LHA are nociceptive and, when activated, attenuate acute and persistent pain.^67,68^ Orexinergic LHA neurons are involved in the induction and maintenance of negative affective-motivational states such as stress and anxiety.^69,70^ Together, molecularly defined excitatory neurons in the LHA with specific axonal targets can explain the effects of LHA^stress-TRAP^ neurons on anxiety levels and itch. In the near future, intersectional genetics^71^ combined with Fos-TRAP techniques will enable us to combine the molecularly defined neuronal population with the stress-sensitive ones in the LHA and test their roles in stress modulation of itch and pain. Molecular profiling of LHA^stress-TRAP^ neurons^72,73^ will shed light on the physiologically relevant neuropeptide or receptor genes expressed in the cell population of our interest and may lead to an understanding of the molecular mechanisms underlying stress modulation of pain and itch.

## Limitations of the study

This study uses RS to induce stress in mice and to investigate the circuit mechanisms underlying the stress-induced modulation of itch. However, it is still unknown whether the LHA-mediated circuits identified here also account for itch modulation triggered by other forms of stress. Additionally, unbiased screening for stress-activated brain regions, followed by mechanistic studies, may uncover neuronal networks outside of the LHA-PAG-RVM pathway that contribute to how stress influences itch.

## Resource Availability

### Lead contact

Requests for further information and resources should be directed to and will be fulfilled by the lead contact, Dr. Arnab Barik (arnabbarik@iisc.ac.in).

### Materials availability

This study did not generate new unique reagents.

### Data and code availability

- Data reported in this paper will be shared by the lead contact upon request.
- This paper does not report original code.
- Any additional information required to reanalyze the data reported in this paper are available from the lead contact upon request.

## Star★Methods

Detailed methods are provided in the online version of this paper and include the following:

- KEY RESOURCES TABLE
- EXPERIMENTAL MODEL AND STUDY PARTICIPANT DETAILS
  ○ Mouse line
- METHOD DETAILS
  ○ Viral vectors
  ○ Antibodies
  ○ Stereotaxic injections
  ○ Fiber-optic cannula and stainless-steel guide cannula implantation
  ○ Stress TRAPping
  ○ Fiber photometry
  ○ Chemogenetic activation
  ○ Optogenetic silencing
  ○ Brain slice preparation and electrophysiology
  ○ Behavioral assays
  ○ Imiquimod induced psoriatic itch
  ○ Hotplate test
  ○ Open field test
  ○ Light-dark box test
  ○ Conditioned Place aversion (CPA) test
  ○ DeepLabCut for tracking mice
  ○ Immunostaining, multiplex *in situ* hybridization, and confocal microscopy
  ○ Input-output mapping of LHA^stress-TRAP^ neurons
- QUANTIFICATION AND STATISTICAL ANALYSIS

## Star★Methods

### Key Resources Table

**Table:** 

REAGENT or RESOURCE	SOURCE	IDENTIFIER
Antibodies		
Chicken anti-GFP antibody	Aveslabs	Catalog# 1010
Phospho-*c*-Fos (Ser32) Rabbit monoclonal antibody	Cell Signaling Technology	Catalog# 5348
Goat anti-tdTomato	SICGEN	Catalog# AB8181
Goat anti-Chicken IgY (H + L) secondary Antibody Alexa Fluor™ 488	Invitrogen	Catalog# A11039
Donkey anti-Rabbit IgG (H + L) secondary Antibody, Alexa Fluor™ 488	Invitrogen	Catalog# A21206
Donkey anti-Goat IgG (H + L) secondary Antibody, Alexa Fluor™ 594	Invitrogen	Catalog# A11058
Bacterial and virus strains		
pAAV5-hsyn-DIO-EGFP	Addgene	Catalog# v78581
pAAV5-FLEX-tdTomato	Addgene	Catalog# 28306
pAAV5-hsyn-DIO-hM3D(Gq)-mCherry	Addgene	Catalog# 44361
rAAV2/9-EF1α-DIO-Kir2.1-P2A-EGFP	BrainVTA	Catalog# PT-1401
AAV9.syn.flex.GcaMP8s	Addgene	Catalog# 162377
rAAV5-EF1α -DIO-oRVG	BrainVTA	Catalog# PT-0023
rAAV5-EF1a-DIO-H2B-eGFP-T2A-TVA	BrainVTA	Catalog# PT-0021
RV-CS-N2C-deltaG-tdTomato	BrainVTA	Catalog# R05002
scAAV-1/2-hSyn1-FLPO-SV40p(A)	University of Zurich	Catalog# v59-1
pAAV-Ef1a-fDIO-tdTomato	Addgene	Catalog# 128434
pAAV5-Ef1a-DIO-eNpHR 3.0-EYFP	Addgene	Catalog# 26966-AAV5
Chemicals, peptides, and recombinant proteins		
4-hydroxytamoxifen	Hello Bio	Catalog# HB0601
Deschloroclozapine (DCZ)	Hello Bio	Catalog# HB9126
Chloroquine	Sigma	Catalog# C6628
Histamine	Sigma	Catalog# H7125
1X PBS	Takara	Catalog# T9181
4% Paraformaldehyde (PFA)	Ted Pella	Catalog# 18505
BSA	HIMEDIA	Catalog# TC194
Triton X-100	SRL	Catalog# 64518
Experimental models: Organisms/strains		
TRAP2 mice (Fos^2A-iCreERT2^)	Jackson Laboratory	Stock number 030323

### Experimental Model and Study Participant Details

#### Mouse line

Animal care and experimental procedures were performed following protocols approved by the CPSCEA at the Indian Institute of Science. TRAP2 (Fos^2A-iCreERT2^)^29^ mice, stock number 030323, were purchased from Jackson Laboratory. The animals were housed at the Central Animal Facility (CAF) under standard transgenic animal housing conditions in a 12-h light-dark cycle with *ad libitum* access to food and water. Genotyping was performed according to the protocols of Jackson Laboratories. CD-1 mice (7–12 weeks old) were purchased from CAF for the behavioral experiments (used in Figure 1). The TRAP2 mice were maintained in the C57BL/6 background. An equal number of males and females underwent stereotaxic surgeries for the behavioral and physiological experiments, unless mentioned otherwise. All mice used in the behavioral assays were between 7 and 12 weeks old. All the behaviors were done during the light cycle.

### Method Details

#### Viral vectors

Vector used and sources: pAAV5-hsyn-DIO-EGFP (Addgene, Catalog# v78581-AAV9, titer-2.5 x 10^13^ GC/mL), pAAV5-FLEX-tdTomato (Addgene, Catalog# 28306-AAV1, titer-1.6 x 10^13^ GC/mL), pAAV5-hsyn-DIO-hM3D(Gq)-mCherry (Addgene, Catalog# 44361, titer-1.8 x 10^13^ GC/mL), rAAV2/9-EF1α-DIO-Kir2.1-P2A-EGFP (BrainVTA, Catalog# PT-1401, titer-2 x 10^12^ vg/ml), AAV9.syn.flex.GcaMP8s (Addgene, Catalog# 162377, titer-2.7 x 10^13^ GC/mL), rAAV5-EF1α-DIO-oRVG (BrainVTA, Catalog# PT-0023, titer-2 x 10^12^ vg/ml), rAAV5-EF1a-DIO-H2B-eGFP-T2A-TVA (BrainVTA, Catalog# PT-0021, titer-2 x 10^12^ vg/ml), and RV-CS-N2C-deltaG-tdTomato (BrainVTA, Catalog# R05002, titer-2 x 10^8^ vg/ml), scAAV-1/2-hSyn1-FLPO-SV40p(A) (University of Zurich, Catalog# v59-1, titer-6.7 x 10^12^ vg/ml), pAAV-Ef1a-fDIO-tdTomato (Addgene, Catalog# 128434-AAV1, titer-1.8 x 10^13^ GC/mL), pAAV5-Ef1a-DIO-eNpHR 3.0-EYFP (Addgene, Catalog# 26966-AAV5, titer-1.1 x 10^13^ GC/mL).

#### Antibodies

Chicken anti-GFP antibody (aveslabs catalog# 1010), Phospho-*c*-Fos (Ser32) Rabbit monoclonal antibody (Cell Signaling Technology Catalog# 5348), Goat anti-tdTomato (SICGEN catalog# AB8181), Goat anti-Chicken IgY (H + L) secondary Antibody, Alexa Fluor 488 (Invitrogen catalog# A11039), Donkey anti-Rabbit IgG (H + L) secondary Antibody, Alexa Fluor 488 (Invitrogen catalog# A21206), Donkey anti-Goat IgG (H + L) secondary Antibody, Alexa Fluor 594 (Invitrogen catalog# A11058).

#### Stereotaxic injections

Mice were anesthetized with 2% isoflurane/oxygen before and during the surgery and mounted on the stereotaxic frame (RWD 69100 Rotational Digital Stereotaxic Frame). An incision was made to expose the skull, and subsequently, the skull was aligned to the horizontal plane. Craniotomy was performed at the marked point using a hand-held micro-drill (RWD). A Hamilton syringe (10 μL) with a glass pulled needle was used to infuse 300 nL of viral particles (1:1 in saline) at a 100 nL/min rate. The following coordinates introduced the virus: LHA-AP: −1.70, ML: ±1.00, DV: −5.15. For rabies tracing experiments, rAAV5-EF1α-DIO-oRVG and rAAV5-EF1α-DIO-EGFP-T2A-TVA were injected first, followed by RV-CS-N2C-deltaG-tdTomato one week after stressTRAPing. Tissue was harvested after 1 week of rabies injection for histochemical analysis. The study did not include the brain tissues where apparent cell death was observed through morphological examination. Post-hoc histological examination of each injected mouse was used to confirm that viral-mediated expression was restricted to the target nuclei.

#### Fiber-optic cannula and stainless-steel guide cannula implantation

Fiber-optic cannulas from RWD (Ø1.25 mm Ceramic Ferrule, 300 μm Core, 0.39NA, L = 7 mm, catalog# R-FOC-BL300C-39NA) were implanted at AP: -1.70, ML: +1.00, DV: -5.15 in the LHA of the AAV-DIO-GCaMP8s-infused mice. The cannulas were fixed to the skull using light-cured dental cement (GC corporation powder-catalog# 002505, liquid-catalog# 002524). Animals were allowed to recover for at least 1 week before performing behavioral tests. Successful labeling and fiber implantation were confirmed post hoc by staining for fluorophores for viral expression and injury caused by the fiber, respectively. Only animals with viral-mediated gene expression and fiber implantations at the intended locations, as observed in post hoc tests, were included in the analysis.

For the chemogenetic activation of LHA^stress-TRAP^ neuron terminal experiments, stainless-steel guide cannulas (O.D. 0.48mm, 26G, 5mm, RWD catalog# 62003) were implanted bilaterally at the PAG (AP: −4.4, ML: ±1.23, DV: −2.86, α = 15°) and PBN (AP: −5.34, ML: 1.00, DV: 3.15) in the stress TRAPed mice. For RVM terminal activation, a8mm cannulas (O.D. 0.48mm, 26G) were implanted at AP: −5.8, ML: 0.10, DV: −5.50 into the RVM of the stress TRAPed mice. The cannulas were fixed to the skull using dental cement, and the animals were allowed to recover for a week before performing behavioral tests. Mice were lightly anesthetized using isoflurane, and the injection tube was inserted into the guide cannula. The infusion cannula was connected to the microinfusion pump (KD Scientific, catalog# 78–8130). Saline/DCZ was infused at the rate of 100 nL/min. The infusion cannula was removed 10 min after the infusion, and 15 min later, behavioral assays were done. Successful implantations were confirmed post hoc by staining for Fos at the intended locations, along with viral expression and injury caused by the cannula. Only animals with viral-mediated gene expression and cannula implantations at the intended locations, as observed in post hoc tests, were included in the analysis.

#### Stress TRAPping

4-hydroxytamoxifen (4-OHT; Hello Bio, UK, Cat No. H6040) was prepared by dissolving it in ethanol at a 20 mg/mL concentration.^29^ The solution was aliquoted and stored at −40°C for several days. 4-OHT was redissolved just before use and mixed with corn oil in a 1:1 ratio. This mixture was vortexed vigorously for 15 min to ensure the suspension of 4-OHT into the corn oil. Next, to remove the residual ethanol, the suspension was held vertically at room temperature for 5 min and spun down at 5000 RPM for 60 s, allowing for phase separation to occur. The supernatant ethanol phase that accumulated at the top of the mixture was then carefully removed via micropieppete. Then the 4-OHT (50 mg/kg body weight) was intraperitoneally administered to the mice, and 15 min later, the mice were subjected to 1-h restraint stress by placing the mouse in a 50 mL Falcon tube, which has holes for proper air ventilation. All the behavioral and anatomical studies were done one week after the stress TRAPing.

#### Fiber photometry

A dual-channel fiber photometry system from RWD (R810) was used to collect the data.^74,75^ The light from two light LEDs (410 and 470 nm) was passed through a fiber-optic cable (RWD-Ø1.25 mm Ceramic Ferrule, 200 μm Core, 0.39NA, L = 2 mm, catalog# R-FC-L-N3-200-L1) coupled to the cannula implanted in the mouse. The fluorescence emission of jRGECO1a was recorded using the RWD R820 fiber photometry system. Fluorescence emission was acquired through the same fiber-optic cable onto a CMOS camera through a dichroic filter. Mice were lightly anesthetized, and the fiber-optic cable was connected to the optical cannula attached to the mouse skull. Mice were habituated to the fibers for 2 days before performing any behavioral assays. The output power was adjusted to 30%, which gives 20–50 μW power at the fiber tip. The signals were acquired at a 30 fps frame rate. The data was analyzed using the RWD photometry software, and.csv files were generated. The start and end of stimuli were timestamped. All trace graphs were plotted from.csv files using GraphPad Prism software version 8.

#### Chemogenetic activation

For chemogenetic activation of LHA^stress-TRAP^ neurons, deschloroclozapine (DCZ) (Hello Bio, catalog# HB9126), 2 μg/kg body weight, was administered intraperitoneally (i.p.) into the stress-TRAPed mice.^76^ All the behavioral assays were done 15 min after the DCZ administration.

#### Optogenetic silencing

For optogenetic inhibition of PAG terminals of the LHA^stress-TRAP^ neurons using halorhodopsin eNpHR3.0,^77^ a bilateral fiber-optic cannula (Ø1.25 mm Ceramic Ferrule, 200 μm Core, 0.22NA, L = 5 mm, catalog# R-FOC-L200C-22NA) was implanted at PAG (AP: −4.4, ML: ±1.23, DV: −2.86, α = 15°). One week after the implantation, mice were habituated for 2 days and then used for behavioral experiments. Prizmatix Optogenetics-LED-Yellow was used to deliver a 595 nm constant light to inhibit the PAG terminals of the LHA^stress-TRAP^ neurons. Mice were briefly anesthetized using isoflurane, and an optical fiber (Prizmatix, L = 2m, core diameter 500 μm, and NA 0.63) was connected to the fiber-optic cannula to deliver light. The scratching behavior of mice was recorded with a 5-min light ON and a 5-min light OFF cycle for 1 h.

#### Brain slice preparation and electrophysiology

TRAP2 mice were injected with AAV encoding Cre-dependent eGFP and, 3 weeks later, stress-TRAPed. One week later, control mice were treated with daily topical application of moisturizer cream (PONDS), while the chronic mice received daily application of imiquimod for 6 days. Control and psoriatic mice were anesthetized with 4% isoflurane, followed by decapitation and surgical dissection of the brain. Coronal brain slices of 300 μm were prepared using semi-automated vibratome (VT1200S; Leica Microsystems, Germany) in ice-cold cutting solutions composed of in mM: sucrose (75), NaCl (87), NaHCO_3_ (25), NaH_2_PO_4_ (1.25), CaCl_2_ (0.5), and MgCl_2_ (7) continuously perfused with carbogen (5% CO2 + 95% O2) gas. The brain slices were placed in a 32°C water bath for 15 min followed by incubation at room temperature (~25°C) for at least an hour in artificial cerebrospinal fluid (ACSF) containing in mM: NaCl (126), KCl (2.5), NaHCO_3_ (25), NaH_2_PO_4_ (1.25), CaCl_2_ (1.5), MgCl_2_ (1.5), and glucose (25) under constant perfusion with carbogen gas, before being considered for patch-clamp experiments. During recording, the brain slices were shifted to a recording chamber bathed with ACSF under carbogen perfusion and maintained at 32°C by using a digitized temperature controller (Warner Instruments, USA).

For whole-cell recordings, the LHA neurons were identified under 40× magnification, displaying eGFP fluorescence emission by using a dot-contrast enabled IR-DIC compatible upright patch clamp microscope (Axio Examiner D1, Carl Zeiss, Germany). Thick-walled borosilicate glass capillaries (OD: 1.5 mm, ID: 0.86 mm) were used for preparing the patch pipettes having 3–5 mΩ resistances by using a horizontal micropipette puller (Sutter Instruments, USA). The patch pipettes were filled with an internal solution composed of in mM: K-gluconate (135), KCl (4), HEPES (10), Na_2_ATP (2), NaGTP (0.5), Na_2_-Phosphocreatine (5), adjusted pH to 7.3 with KOH. Whole-cell current clamp experiments were performed using a computer-controlled Multiclamp 700B amplifier operated through pClamp 11.3 software (Molecular Devices, USA). The current and voltage traces were low-pass filtered with 2 kHz and digitized at 10 kHz using a hum-silencer-enabled Digidata 1550B (Molecular Devices, USA). The resting membrane potential was noted immediately after attaining whole-cell mode without any external current injection. Postsynaptic step current injections of 3 s, ranging from 0 to +200 pA with 20 pA current increments, were used to assess the gain of firing rate (F/I plot) of LHA neurons. Similarly, for determining the input resistance, −100 to +100 pA postsynaptic current was injected through a patch pipette with a 10-pA increment. All the electrophysiological data were analyzed using the Clampfit module of pClamp software, and Adobe Illustrator was used to plot the graphs.

### Behavioral assays

#### Chloroquine and histamine-induced itch assay

The nape of the neck of mice was shaved with a hand-held Philips shaver 2–3 days before behavioral experimentation, and the mice were habituated in the behavior room. Unless otherwise stated, the mice used for behavioral studies were blinded prior to initiation of the studies by an individual not involved in the experimentations described here. All behavioral experiments were quantified by one experimenter and randomly cross-verified by another. All itch experiments were videotaped with a Logitech camera, and videos were acquired through vendor-supplied software. Mice were individually placed in four-part plexiglass chambers with chamber dimensions of 6 cm × 6 cm × 14 cm. The roof of the chamber had holes for air ventilation. Animals were habituated in the chamber for 15 min before chloroquine injections. DCZ 2 μg/kg body weight was administered intraperitoneally (i.p.) 15 min before chloroquine injection. Chloroquine (375 μg/75 μL) (Sigma Catalog# C6628) or histamine (500 μg/50 μL) (Sigma Catalog# H7125) was administered intradermally into the nape of the neck of the mice, and the subsequent scratching behavior was recorded for 30 min.^56^ Hind leg-directed scratching of the nape was characterized as a scratch, and the videos were quantified, blinded to the experimental conditions.

#### Imiquimod induced psoriatic itch

Imiquimod (5% w/w from Glenmark) was used to induce psoriasis in mice.^78,79^ To induce psoriasis-like chronic itch, the nape of each mouse was shaved using Veet hair removal cream, and imiquimod was topically applied once daily to the shaved area for six consecutive days. This treatment reliably induced inflamed, scaly skin lesions characterized by thickened and dry epidermis. Mice were manually inspected, and only those exhibiting pronounced psoriatic features—namely, inflamed, thickened, and scaly skin—were selected for behavioral analysis. Following psoriasis induction, mice were individually placed in four-compartment plexiglass chambers for habituation, after which their behavior was recorded for 30 min. Spontaneous scratching was defined as hind limb-directed contact to the nape region. Scratching bouts were quantified by observers blinded to the experimental conditions.

#### Hotplate test

The thermal hotplate experiments were performed using the Hot and Cold Plate analgesiometer (HC-01, Orchid Scientific).^36,80^ The specifications of the instrument used were-enclosure size: 205 × 205 × 250 mm; plate size: 190 × 190 × 06 mm; temperature range: −5°C to 60°C. A single experimenter introduced the mice into the enclosure on the thermal plate across all the experiments and performed analysis in a blinded manner. The mice were habituated in the experimental room for 30 min and in the enclosure for 5 min at 32°C before the experimentation for three consecutive days. On the experimental day, mice were placed on the hotplate at 52°C, and the behavior was recorded for 45 s using three Logitech web cameras placed at the left, right, and front angles around the hotplate.^80^

Later, videos were quantified individually for any nocifensive behaviors (licks, shakes, and jumps) exhibited by the mice, blinded to the experimental conditions.

#### Open field test

The open field test was used to evaluate anxiety-like behavior.^81^ The open field arena was made up of acrylic white opaque walls with dimensions 50 cm (length) x 50 cm (width) x 38 cm (height). The central field has a dimension of 30 cm × 30 cm. The mouse was placed in the middle of the arena and allowed to move freely for 10 min. The movement was recorded using an overhead-mounted Logitech camera. The open field arena was cleaned with 70% alcohol between every trial. The total distance moved and the time spent in the central field was tracked using DeepLabCut.

#### Light-dark box test

The light-dark box with dimensions 40 cm (length) x 20 cm (width) x 36 cm (height) of each chamber (light and dark chamber) was used to measure the anxiety-like behavior.^82,83^ Mice could freely access both the light and dark chambers via a small opening that connected them. The mice were introduced into the light side of the apparatus and allowed to explore freely for 15 min, and the movement was recorded using a Logitech camera (C930e) from the top. The time spent in the light box taken as the degree of anxiety in the mouse. The apparatus was cleaned with 70% alcohol between every trial. The movement of the mouse was tracked and plotted using DeepLabCut.

#### Conditioned Place aversion (CPA) test

A three-compartment custom-built CPA apparatus was used to test the conditioned place aversion in mice. Both outer chambers have a dimension of 32 cm (length) x 32 cm (width) x 28 cm (height), while the middle chamber has a dimension of 11 cm × 8.5 cm. One of the outer chambers has white stripe walls and a steel mesh floor; the other has black walls and a steel rod floor; the middle has gray walls and a smooth PVC floor as a neutral zone. Two manual doors between these three chambers can be closed to block entry into any of the chambers. The light intensity was constant to prevent any innate preference. Each chamber was cleaned thoroughly with 70% ethanol between every trial. The mouse movement was videotaped using a Logitech camera (C930e).

The unbiased CPA experiment ends in 5 days.^84^ On Day 1 (pre-conditioning phase), mice were placed in the central compartment of a three-chamber apparatus and allowed to explore all chambers for 15 min. The time spent in each outer chamber (T1 and T2) was recorded. Mice exhibiting a preference ratio (T1/T2) between 2:3 and 3:2 were selected for further experiments; animals outside this range were excluded to ensure unbiased baseline preferences. From Days 2–4 (conditioning phase), animals underwent two daily conditioning sessions (morning and evening), separated by a minimum interval of four hours. On Day 2, the chamber with striped walls was designated as the drug-paired environment (DCZ), while the chamber with black walls was designated as the saline-paired environment. In the morning session, mice received an intraperitoneal (i.p.) injection of DCZ and were confined to the drug-paired chamber for 30 min. In the evening session, they received an i.p. injection of saline and were confined to the saline-paired chamber for 30 min. On Day 3, the conditioning sequence was reversed: mice received saline in the morning and DCZ in the evening, with corresponding chamber confinement. On Day 4, the original sequence was reinstated, with DCZ administered in the morning and saline in the evening. On Day 5 (post-conditioning phase), both chamber doors were opened, and mice were allowed to explore the entire apparatus freely for 15 min. Time spent in the DCZ-paired chamber was compared between Day 1 and Day 5 to evaluate the development of conditioned place aversion (CPA).

#### DeepLabCut for tracking mice

The tracking of mice in the open field test, light-dark box test, and CPA test was done using DeepLabCut (DLC) (version 2.3.9).^80,85^ Data were processed and analyzed on a custom-built workstation equipped with an AMD Ryzen 9 5900X 12-core processor and an NVIDIA GPU. For training the DeepLabCut (DLC) model, 20 frames were manually labeled from each of the five videos. Following training, the model was used to analyze the videos, generating position plots and corresponding output in.csv format. For visualization, representative plots of tracked spine positions were produced.

#### Immunostaining, multiplex *in situ* hybridization, and confocal microscopy

Mice were anesthetized with isoflurane and perfused transcardially with 1× phosphate buffered saline (PBS) (Takara catalog# T9181) and 4% Paraformaldehyde (PFA) (Ted Pella, Inc. catalog# 18505), harvested brains and spinal cords were further fixed in 4% PFA, overnight, and subsequently transferred to 15% and 30% sucrose for serial dehydration. Brain tissues were placed in the Cryo-Embedding Compound (Ted Pella, Inc.) and frozen at −40°C. Subsequently, 50 μm-thick coronal brain sections were cut using a cryostat (RWD Minux FS800). For immunostaining experiments, tissue sections were rinsed in 1× PBS (3 times) and incubated in the blocking buffer (5% Bovine Serum Albumin (BSA) + 0.5% Triton X-100 + 1× PBS) (BSA-HIMEDIA catalog# TC194, Triton X-100 SRL catalog# 64518) for one hour at room temperature. Sections were then incubated in the primary antibody (dilution 1:1,000× in blocking buffer) at room temperature overnight (not more than 12–14 h). Sections were rinsed 3 times with 1× PBS +0.5% Triton X-100 solution and incubated for two hours in Alexa Fluor conjugated goat anti-rabbit/chicken or donkey anti-goat/rabbit secondary antibodies (dilution 1:1,000× in blocking buffer) along with DAPI (SRL catalog# 18668) at room temperature. Then sections were washed with 1× PBS +0.5% Triton X-100, and mounted onto charged glass slides (Ted Pella, Inc. catalog# 260382-3). Citifluor AF-1 mounting media (Ted Pella, Inc. catalog# 19470-1) was used to coverslip (Blue star microscopic cover glass 24 × 60 mm 10 Gms) the slides. Subsequently, sections were imaged on the upright fluorescence microscope (Khush Enterprises, Bengaluru) (2×, 4×, and 10× lenses) and a Confocal Microscope (Leica SP8 Falcon, Germany). ImageJ/FIJI processing software was used to process the images. Confocal images were processed using the Leica image analysis suite software.

Fresh brains were rapidly harvested and flash-frozen at −80°C for subsequent *in situ* hybridization (ISH). Coronal sections (20 μm) were prepared using a cryostat. Multiplex ISH was performed using the manual RNAscope assay (Advanced Cell Diagnostics, ACD). Target-specific probes were obtained from the ACD online catalog: *Slc17a6* (Ref. #319171), *tdTomato* (Ref. #317041), and *Slc32a1* (Ref. #319191). Frozen brain sections were fixed in 4% paraformaldehyde (PFA; Ted Pella, Inc., Cat. #18505) for 15 min at room temperature, followed by sequential dehydration in graded ethanol (Hayman, Cat. #64-17-5) for 20 min. After brief air drying, a hydrophobic barrier was drawn around each tissue section. RNAscope Hydrogen Peroxide (Cat. #322335) was applied for 10 min, followed by two rinses in nuclease-free water (MP Biomedicals, Cat. #112450204). Sections were then incubated with Protease IV (Cat. #322336) for 30 min and rinsed twice in nuclease-free water. A mixture of probes was prepared at a ratio of 50:1:1 for *Slc17a6* (channel 1), *tdTomato* (channel 2), and *Slc32a1* (channel 3), and applied to the sections. Hybridization was carried out for 2.5 h at 40°C using the HyperChrome hybridization system (HyperChrome, Cat. #EHP 500AS). Following hybridization, sections were washed with 1× wash buffer (Cat. #320058), and signal amplification and chromogenic development were performed according to the manufacturer’s protocol (ACDBIO). Images for anatomical analysis were acquired using 10× and 20× objectives on a Leica SP8 Falcon laser scanning confocal microscope (Leica Microsystems, Germany) and processed using Leica image analysis suite software.

#### Input-output mapping of LHA^stress-TRAP^ neurons

To map the brain-wide monosynaptic inputs of the LHA^stress-TRAP^ neurons, we used the pseudorabies virus-based retrograde tracing strategy.^86,87^ Briefly, rAAV5-EF1α-DIO-oRVG and rAAV5-EF1α-DIO-EGFP-T2A-TVA were injected first. Three weeks later, mice were stress-TRAPed as described above, and then a second injection of RV-CS-N2C-deltaG-tdTomato was done one week after the stress-TRAPing. Brain tissue was harvested after 1 week of rabies injection for histochemical analysis as described above. Every 3rd brain section was mounted and imaged on the upright fluorescence microscope (Khush Enterprises, Bengaluru) (2×, 4×, and 10× lenses). ImageJ/FIJI processing software was used to process the images, and the number of tdTomato-positive cells was counted and reported from 3 mice.

To map the brain-wide downstream targets of the LHA^stress-TRAP^ neurons, AAV-DIO-tdTomato was injected into the LHA of the TRAP2 mice, and stress-TRAPing was done 3 weeks after the injection. One week after the stress-TRAPing, the brain tissue was harvested, and the brain-wide projections of the LHA^stress-TRAP^ neurons were imaged under fluorescent microscopy. Gain and exposure time were kept constant throughout the imaging session. A box area was selected in the region containing the tdTomato-positive fibers to calculate the density of the projection, and the mean intensity (F_Total_) was calculated using ImageJ/FIJI. To calculate the background intensity (F_Background_), the same box area was dragged to a region where tdTomato-positive fibers were absent on the same brain section, and then the mean intensity was calculated. The mean fluorescent intensity of the region of interest (F_ROI_) was reported as F_ROI_ = F_Total_ - F_Background_.

### Quantification and Statistical Analysis

All statistical analyses were performed using GraphPad PRISM 8.0.2 software. Student *t* test and two-way ANOVA tests were performed wherever applicable. ns > 0.05, * *p* ≤ 0.05, ** *p* ≤ 0.01, *** *p* ≤ 0.001, **** *p* ≤ 0.0005.

## Supplemental information

Supplemental information can be found online at https://doi.org/10.1016/j.celrep.2026.117025.

## Highlights

- LHA neurons mediate stress modulation of itch
- LHA neurons are sufficient and necessary for stress-induced itch suppression
- LHA neurons are potentiated by chronic psoriatic itch Stress-sensitive
- LHA neurons mediate itch through downstream PAG neurons
